# Peptidase PepP is a novel virulence factor of *Campylobacter jejuni* contributing to murine campylobacteriosis

**DOI:** 10.1080/19490976.2020.1770017

**Published:** 2020-06-25

**Authors:** Markus M. Heimesaat, Anna-Maria Schmidt, Soraya Mousavi, Ulrike Escher, Nicole Tegtmeyer, Silja Wessler, Gabriele Gadermaier, Peter Briza, Dirk Hofreuter, Stefan Bereswill, Steffen Backert

**Affiliations:** a Institute of Microbiology, Infectious Diseases and Immunology, Gastrointestinal Microbiology Research Group, Charité - University Medicine Berlin, Corporate Member of Freie Universität Berlin, Humboldt-Universität Zu Berlin, And Berlin Institute of Health, Berlin, Germany; b Division of Microbiology, Department of Biology, Friedrich Alexander University Erlangen/Nuremberg, Erlangen, Germany; c Department of Biosciences, Paris Lodron University of Salzburg, Salzburg, Austria; d Department of Biological Safety, German Federal Institute for Risk Assessment (Bfr), Berlin, Germany

**Keywords:** Campylobacteriosis, secondary abiotic IL-10^−/-^ mouse model, pro-inflammatory immune responses, M24 peptidase family, Xaa-Pro aminopeptidase, pepP, host–pathogen interaction

## Abstract

Mechanisms of host–pathogen interactions resulting in immunopathological responses upon human *Campylobacter jejuni* infection are not completely understood, but the recent availability of murine infection models mimicking key features of campylobacteriosis helps solving this dilemma. During a screen for proteases expressed by *C. jejuni*, we identified a peptidase of the M24 family as a potential novel virulence factor, which was named PepP. The gene is strongly conserved in various *Campylobacter* species. A constructed deletion mutant Δ*pepP* of *C. jejuni* strain 81–176 grew as efficiently compared to isogenic wild-type (WT) or *pepP* complemented bacteria. To shed light on the potential role of this protease in mediating immunopathological responses in the mammalian host, we perorally challenged microbiota-depleted IL-10^−/-^ mice with these strains. All strains stably colonized the murine gastrointestinal tract with comparably high loads. Remarkably, *pepP* deficiency was associated with less severe induced malaise, with less distinct apoptotic and innate immune cell responses, but also with more pronounced proliferative/regenerative epithelial cell responses in the large intestine at d6post-infection. Furthermore, pro-inflammatory mediators were lower in the colon, ileum, and mesenteric lymph nodes of mice that had been challenged with the Δ*pepP* mutant compared to the WT or *pepP* complemented strains. This also held true for extra-intestinal organs including liver, kidneys, and lungs, and, strikingly, to systemic compartments. Taken together, protease PepP is a novel virulence determinant involved in mediating campylobacteriosis. The finding that apoptosis in the colon is significantly diminished in mice infected with the *pepP* mutant highlights the epithelial layer as the first and main target of PepP in the intestine.

## Introduction

Foodborne diseases caused by *Campylobacter, Salmonella*, pathogenic *Escherichia coli*, and other enteropathogenic bacterial species represent significant public health burdens, responsible for high rates of morbidity and mortality, especially in children.^1^ Of these pathogens, *Campylobacter jejuni* is responsible for approximately 96 million enteritis cases in humans worldwide.^2^ The natural niche of *C. jejuni* is the avian intestine, where the bacteria reside as commensals, but in mammals and especially in humans they may cause gastroenteritis. The pathogen enters the food chain via contaminated animal products, and consumption of contaminated poultry meat is a major recognized risk factor.^3^ Typically, following oral uptake in humans, *C. jejuni* colonizes the mucus layer of the large intestine. While the infection can remain asymptomatic, possibly related to the immune status and low-dose or regular exposure,^4^ the majority of sporadic exposure incidents result in symptoms ranging from mild, self-limiting diarrhea to severe inflammatory bloody diarrhea, often accompanied by fever and abdominal pain. This clinical manifestation of campylobacteriosis is practically indistinguishable from salmonellosis.^5^ Infections with *C. jejuni* are potentially also associated with serious sequelae, including Guillain–Barré syndrome, irritable bowel disease, and reactive arthritis.^6^

Multiple studies have reported that various bacterial factors play a role in the pathogenesis of *C. jejuni* and these have been reviewed on multiple occasions.^7–12^ The literature mostly agrees that bacterial adhesion to intestinal epithelial cells and subsequent cell entry provides the primary cause of tissue damage in the human host. Intimate attachment of *C. jejuni* is facilitated by specialized outer membrane proteins that function as adhesins, although their identification has in part resulted in conflicting results.^13,14^ These include *Campylobacter* adhesion to fibronectin (CadF), fibronectin-like protein A (FlpA), periplasmic binding protein (PEB1), major outer membrane protein (MOMP), *C. jejuni* lipoprotein A (JlpA), *Campylobacter* autotransporter protein A (CapA), and p95.^15,16^ The relative importance of the various adhesins identified so far, their interplay with host factors and subsequent consequences to pathogenesis are still much debated and at the time remain only poorly understood. Using *in vitro* models of epithelial cell invasion, it was observed that non-motile flagellar mutants of *C. jejuni* exhibit significant deficiencies to enter cells.^17,18^ It seems likely that flagella-driven motility enables the bacteria to propel themselves into the cells, while the flagellum can also serve as a type III secretion system (T3SS) to deliver virulence factors into the host cell.^19^ Various secreted substrates were described including the *Campylobacter* invasion antigens (CiaB, CiaC, and CiaD) and flagellar co-expressed determinant (Fed) protein, all of which may trigger host cell entry.^20–22^ In addition, secreted *C. jejuni* proteases are now recognized to be involved in virulence.

Recently we have shown that secreted serine protease high-temperature requirement A (HtrA) assists in bacterial invasion and transmigration.^23,24^ The protein helps *C. jejuni* to cross the epithelial barrier through paracellular translocation by cleaving tight junction proteins, such as occludin and E-cadherin.^25^ However, *C. jejuni* also encodes other proteases that may exhibit fundamental functions in the infection process. Over 45 peptidase-related proteins have been identified by *in silico* analysis of the *C. jejuni* NCTC11168 genome,^26^ although their possible roles in pathogenesis remain to be established experimentally. When we applied the casein zymography assay to reveal the molecular weight of HtrA monomers and oligomers,^27^ we noted the presence of another active protease that migrated at about 70 kDa. Here, we aimed to identify and characterize this novel protease in detail. After identification of its coding gene, we constructed a gene deletion mutant and compared the virulence potential of isogenic parental and mutant strains to assess a possible role in virulence in an established murine *C. jejuni* infection model. We could recently show that upon depletion of the gut microbiota by broad-spectrum antibiotic treatment, IL-10^−/-^ mice could not only be stably infected with high *C. jejuni* loads, but also developed acute, non-self-limiting *C. jejuni* induced enterocolitis within 1 week post-infection (p.i.), thereby mimicking key features of severe human campylobacteriosis.^28^ Our present *in vivo* study revealed that the 70 kDa protease, identified as a member of the M24 peptidase family, constitutes an important *C. jejuni* virulence factor mediating intestinal, extra-intestinal and even systemic immunopathological sequelae of mammalian *C. jejuni* infection.

## Results

### Identification of *C. jejuni* PepP peptidase exhibiting proteolytical activity

**Figure 1.**
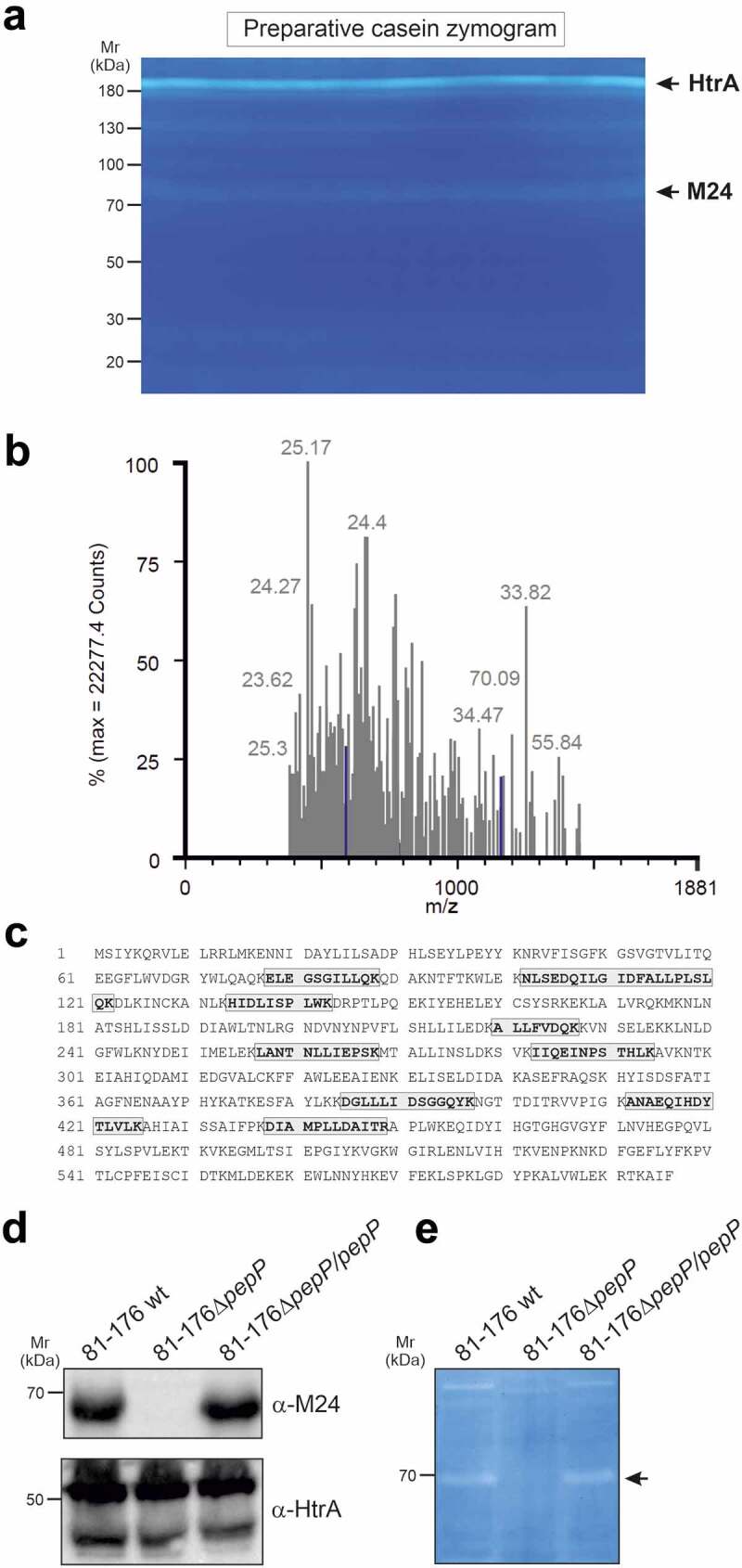
Identification of *C. jejuni* proteins exhibiting proteinolytical activity.

To identify the major proteases produced by *C. jejuni*, total bacterial lysate of strain 81–176 was separated on a preparative SDS-PAGE gel containing embedded casein. After protein renaturation by removal of SDS, zymography demonstrated proteolytic activity of one major and several weaker protein bands (Figure 1a). The main band of 180 kDa corresponded to oligomeric forms of HtrA, as we had demonstrated previously.^27^ The weaker band with mobility of approximately 70 kDa was excised and following trypsin treatment peptides were analyzed by mass spectrometry (Figure 1b). Of the detected peptide sequences, nine were identified as identical to a peptidase of the M24 family described as UniProtKB protein A0A0M3VCY5 (locus WP_002854975) (Figure 1c). The corresponding gene in the genome sequence of *C. jejuni* 81–176 (NC_008787.1), with locus tag CJJ81176_0681, is annotated as ‘aminopeptidase P family protein’. The protein is highly conserved in other *C. jejuni* strains and contains three conserved Pfam domains, PF01321 und PF16189, typical for creatinase/prolidase activity, and PF16188, the C-terminal region of peptidase M24 (Supplementary Figure S1). Based on this information, the translated protein most likely represents a protease (E.C. 3.4.11.9) that catalyzes the release of any N-terminal amino acid that is linked with proline. This enzyme is a member of the MEROPS peptidase M24 family, and is possibly an Xaa-Pro aminopeptidase. The corresponding gene was named *pepP*. This gene is not only highly conserved in *C. jejuni*; a homolog was also identified in *C. coli, C. hepaticus, C. upsaliensis*, and *C. helveticus*, and a distant homolog was present in *C. cuniculorum*, but it was not detected in other *Campylobacter* species. The closest homolog in an alternative genus was identified in *Helicobacter mesocricetorum* as shown in a phylogenetic tree (Supplementary Figure S2).

*C.jejuni* lysate was analyzed by zymography on apreparative scale (a). Negatively stained protease bands were excised and analyzed by mass-spectrometry (RMS error 32.6650%) (b). Amino acid sequence of PepP peptidase (UniProtKB A0A0M3VCY5, formerly E6RX75_CAMJC) with the identified peptides highlighted with gray boxes (sequence coverage 18.9%, MW 68.3 kDa) (c). Expression of PepP with HtrA as control was confirmed by Western blotting using indicated antibodies (d) and proteolytic activity was investigated by casein zymography (e).

### PepP deficiency does not compromise the intestinal colonization of *C. jejuni* in IL-10^−/-^ mice

**Figure 2.**
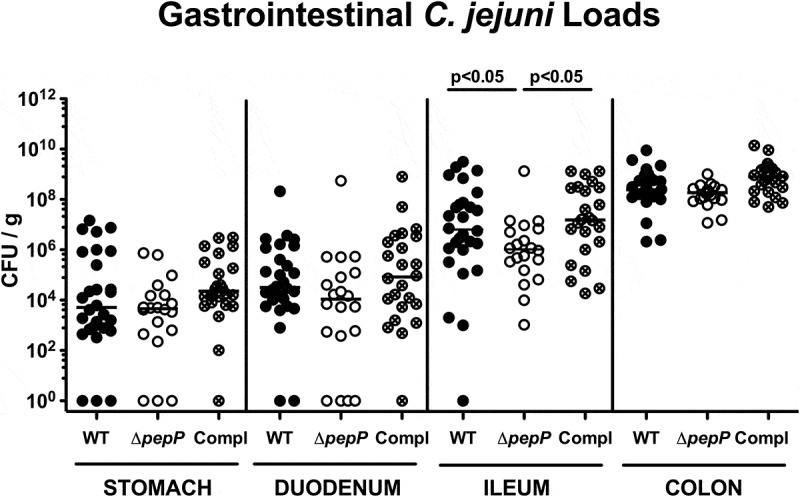
Gastrointestinal *C. jejuni* loads in infected mice.

A knockout mutant of *pepP* was constructed by homologous gene recombination using a construct in which the gene was interrupted by a kanamycin gene. The knockout mutant was checked by PCR and subsequent amplicon sequence analysis. Genetic complementation of the ∆*pepP* mutant with the WT *pepP* gene was performed as described previously.^24^ Presence or absence of PepP expression and proteolytical activity was confirmed by Western blotting using generated α-M24 (PepP) antibodies (Figure 1d) and casein zymography (Figure 1e), respectively. No significant growth differences were observed between the deletion mutant and its parental WT strain when grown on rich media (Supplementary Figure S3). Next, the knockout mutant and the isogenic WT strain were tested in the IL-10^−/-^ mouse infection model.^28^ Therefore, microbiota-depleted IL10^−/-^ mice were perorally infected with approximately 10^9^ CFU of the *C. jejuni* Δ*pepP*, the *pepP* complemented or the WT strain on d 0 and 1 by gavage. Daily cultural analysis of fecal samples revealed high median bacterial loads of approximately 10^9^ colony forming units (CFU) per g from d 2 until d 6 p.i., without differences between the three tested strains (Supplemental Figure S4). Likewise, the postmortem luminal loads on d 6 in the stomach, duodenum, and colon were comparable between respective strains (n.s.; Figure 2), whereas ileal bacterial numbers were slightly, but significantly lower in mice that had received the Δ*pepP* mutant as compared to the WT or pepP complemented strains (*p* < .05) (Figure 2). Hence, inactivation of the *pepP* gene did not compromise the colonization properties of *C. jejuni* in the colon, duodenum, and stomach, but decreased this ability in the ileum following peroral infection of microbiota-depleted IL-10^−/-^ mice.

Microbiota-depleted IL-10^−/-^ mice were perorally infected either with the *C.jejuni* 81–176 wild-type strain (WT; black circles, n= 18), the isogenic *pepP* gene deletion mutant (Δ*pepP*; white circles, n= 20) or the *pepP* gene complemented strain (Compl; crossed circles, n= 24) on d0 and d1. *C.jejuni* loads were determined in luminal samples taken on d6 post-infection by culture and expressed as colony-forming units per g(CFU/g) for stomach, duodenum, ileum, and colon. Medians (black bars) and levels of significance (*p*-values) assessed by the Mann–Whitney Utest are indicated. Data were pooled from four independent experiments.

### Clinical symptoms are less severe upon *pepP* inactivation

**Figure 3.**
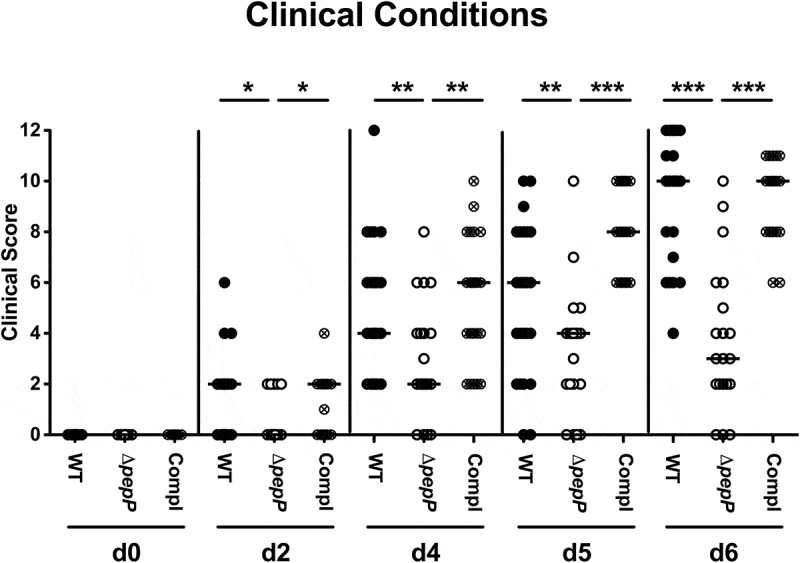
Time course of disease development in *C. jejuni* infected mice.

The clinical conditions of the infected animals differed significantly depending on the applied strain. As early as d2 post-infection the mice that had received the Δ*pepP* mutant displayed significantly lower clinical scores compared to WT strain or *pepP* complemented strain-infected counterparts (*p* < .05–0.001; Figure 3). The strongest difference was observed on d6 p.i., when both, WT and *pepP* complemented strain infected mice were suffering from acute enterocolitis as indicated by wasting and bloody diarrhea mounting in a median clinical score of 10, whereas Δ*pepP-*infected mice displayed variable but mostly mild disease resulting in a median clinical score of 3 (*p* < .001; Figure 3). Hence, *pepP* gene deficiency was associated with an impaired ability of *C. jejuni* to induce disease in the here applied murine model.

Clinical scores were assessed prior to challenge and during the infection experiment with the *C.jejuni* 81–176 wild-type strain (WT; black circles, n= 18), the Δ*pepP* mutant (white circles, n= 20) or *pepP* complemented strain (Compl; crossed circles, n= 24). Clinical scores were assessed on adaily basis (as indicated; d,day) applying astandardized clinical scoring system. Medians (black bars) and significance levels (*p*-values; * *p*< .05; ** *p*< .01; *** *p*< .001) as determined by the Mann–Whitney Utest are indicated. Data were pooled from four independent experiments.

### Microscopic inflammatory sequelae are less severe upon Δ*pepP* inactivation

**Figure 4.**
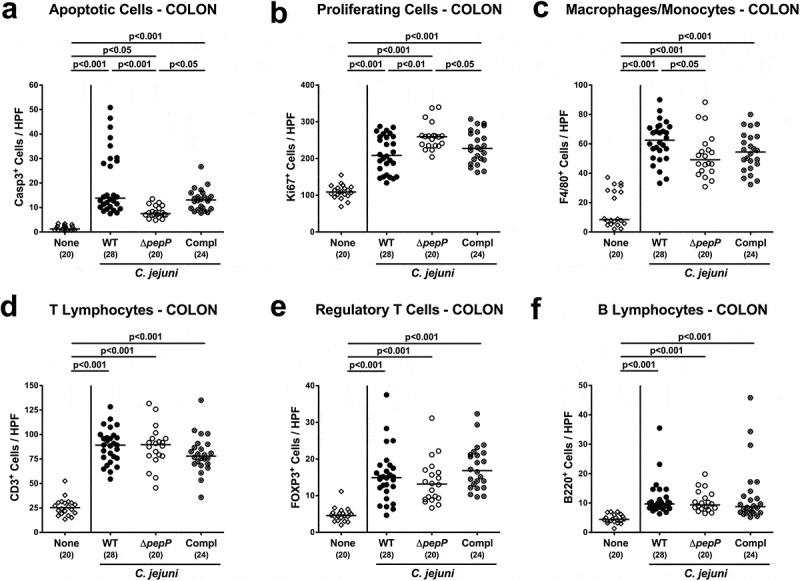
Apoptotic, proliferative/regenerative, and immune cell responses in the colon.

We further assessed microscopic changes of the colonic mucosa as a result of *C. jejuni* infection, for which we determined the numbers of caspase3^+^ apoptotic epithelial cells and of Ki67^+^ proliferating/regenerating epithelial cells. At necropsy, challenged mice displayed increased numbers of either cell type in their colonic mucosa as compared to uninfected control animals (*p* < .001; Figure 4a,b; Supplemental Figure S5A,B). Mice infected with Δ*pepP* exhibited lower numbers of apoptotic cells, but higher Ki67^+^ cell counts in their large intestinal mucosa when compared to WT and *pepP* complemented strain-infected counterparts (*p* < .05–0.001; Figure 4a,b; Supplemental Figure S5A,B), indicative of more pronounced regenerative measures counteracting the pathogen-induced cell damage.

Shown are average numbers obtained from the colon of *C.jejuni* 81–176 WT (black circles), Δ*pepP* (white circles) or *pepP* complemented strain (Compl; crossed circles) infected mice at d6 post-infection for (a) apoptotic cells that are positive for caspase-3, (b) proliferating/regenerating cells positive for Ki67, (c) macrophages and monocytes positive for F4/80, (d) CD3^+^ Tlymphocytes, (e) FOXP3^+^ regulatory Tcells, and (f) B220^+^ Blymphocytes. All data are obtained from six high power fields (400x magnification) per mouse. Uninfected control mice received vehicle only (none, white diamonds). Medians (black bars), significance levels (*p*-values) determined by the one-sided ANOVA test and Tukey post-correction or the Kruskal–Wallis test and Dunn’s post-correction and the numbers of analyzed mice (in parentheses) are indicated. Data were pooled from four independent experiments.

We also investigated *C. jejuni* induced colonic immune cell responses by quantification of distinct innate and adaptive immune cell subsets applying *in situ* immunohistochemistry. *C. jejuni* infection was associated with an increase in innate immune cell populations such as F4/80+ macrophages and monocytes, which was also true for adaptive immune cell subsets including CD3^+^ T lymphocytes, FOXP3^+^ regulatory T cells, and B220^+^ B lymphocytes (*p* < .001; Figure 4c–f; Supplemental Figure S5 C-F). Interestingly, only numbers of macrophages and monocytes (*p* < .05), but not of the analyzed adaptive immune cell subsets were lower in the colonic mucosa and lamina propria following Δ*pepP* as compared to the WT strain infection (Figure 4c; Supplemental Figure S5 C). Hence, inactivation of the *pepP* gene is associated with less *C. jejuni* induced apoptotic and innate immune cell responses, whereas more pronounced proliferative and regenerative cell measures were observed in the large intestinal tract of the Δ*pepP* challenged mice.

### Deletion of *pepP* results in weaker pro-inflammatory mediator secretion

**Figure 5.**
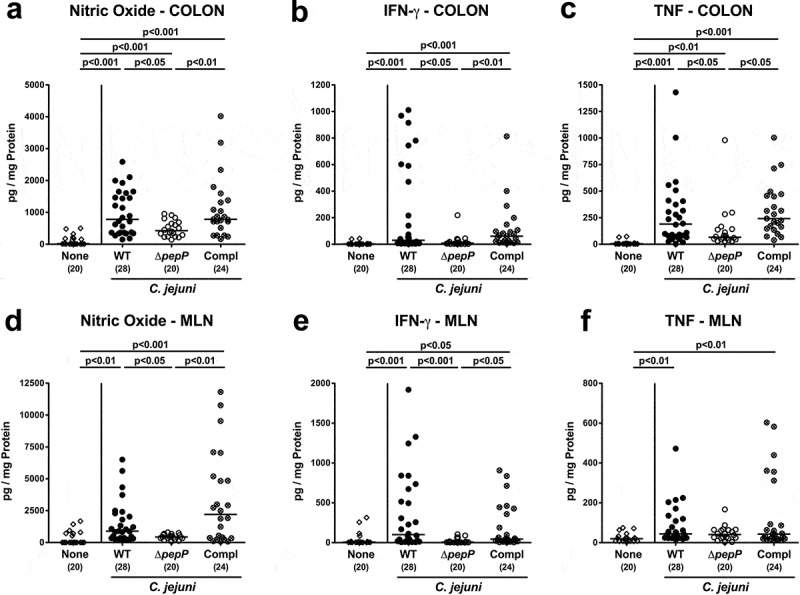
Secreted intestinal pro-inflammatory mediators in *C. jejuni* infected mice.

We further surveyed pathogen-induced secretion of pro-inflammatory mediators in distinct parts of the intestinal tract. Colonization with either strain resulted in increased concentrations of nitric oxide and TNF in the colonic biopsies (*p* < .01–0.001; Figure 5a,c), which also held true for colonic IFN-γ secretion upon WT strain or *pepP* complemented strain infection (*p* < .001; Figure 5b). Respective mediators were, however, lower upon Δ*pepP* versus WT or *pepP* complemented strain infection (*p* < .05–0.01; Figure 5a–c). Remarkably, infection with WT and *pepP* complemented strains, but not with Δ*pepP* resulted in increased concentrations of nitric oxide, IFN-γ, and TNF in mesenteric lymph nodes (MLN) (*p* < .05–0.001; Figure 5d–e), and similar differences were obtained for IFN-γ and TNF levels in ileal biopsies (*p* < .01–0.001; Supplemental Figure S6). Hence, *pepP* gene deficiency was associated with weaker *C. jejuni-*induced pro-inflammatory mediator secretion in both the distal small and large intestines.

Quantitative analytical data obtained from colon (top) and mesenteric lymph nodes (MLN, bottom) of mice infected with WT (black circles), Δ*pepP* (white circles) or *pepP* complemented strain (Compl; crossed circles) for (a, d) nitric oxide, (b, e) IFN-γ, and (c, f) TNF concentrations. These were measured in supernatants of *ex vivo* biopsies extracted on d6 post-infection. Uninfected controls received vehicle only (none, white diamonds). Medians (black bars), significance levels (*p*-values) determined by the Kruskal–Wallis test and Dunn’s post-correction and the numbers of analyzed mice (in parentheses) are indicated. Data were pooled from four independent experiments.

**Figure 6.**
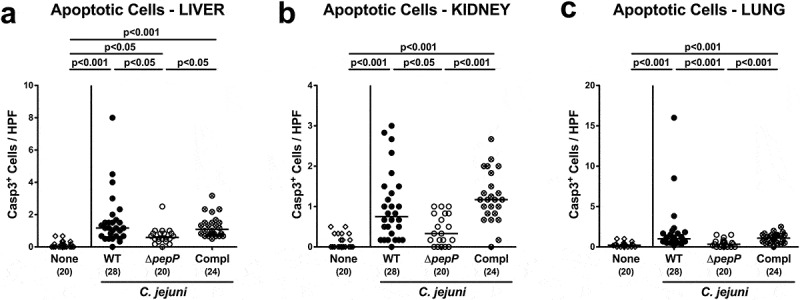
Apoptotic and proliferative/regenerative cells in extra-intestinal organs.

**Figure 7.**
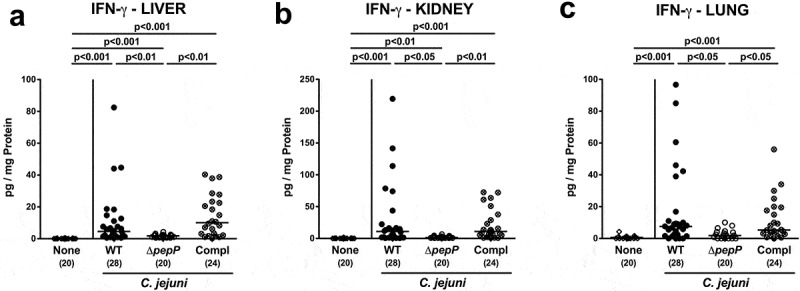
IFN-γ secretion in extra-intestinal organs.

**Figure 8.**
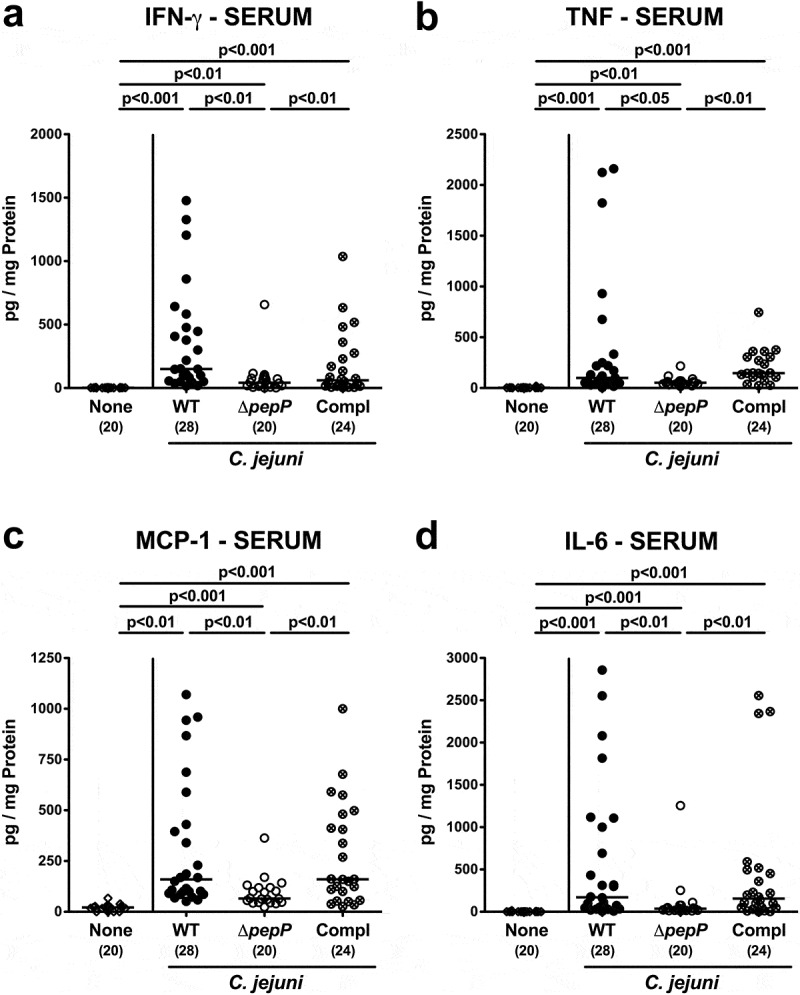
Systemic pro-inflammatory mediator secretion.

Interestingly, the observed *C. jejuni* strain-dependent inflammatory effects were not restricted to the intestinal tract, as they could also be observed in other organs (Figure 6; Supplemental Figure S7). Mice infected with Δ*pepP* exhibited lower numbers of apoptotic cells in liver, kidneys, and lungs as compared to WT strain and *pepP* complemented strain-infected counterparts (*p* < .05–0.01; Figure 6a–c; Supplemental Figure S7A-C) that were accompanied by less distinct pro-inflammatory IFN-γ secretion in respective extra-intestinal compartments of the former versus the latter two control cohorts (*p* < .001 and *p* < .01, respectively; Figure 7). Strikingly, even systemic *C. jejuni* induced pro-inflammatory cytokine responses were dependent on an intact *pepP* gene, given that IFN-γ, TNF, MCP-1, and IL-6 concentrations were lower in serum samples following Δ*pepP* versus WT and *pepP* complemented strain infection (*p* < .05–0.01; Figure 8). Hence, an intact *pepP* gene is not only required to induce campylobacteriosis in the intestinal tract of the challenged mice, but also for eliciting a pro-inflammatory response in extra-intestinal organs and even for systemic responses.

(a) Liver, (b) kidney, and (c) lung biopsies obtained 6 dfollowing infection with *C.jejuni* 81–176 WT (black circles), Δ*pepP* (white circles) or *pepP* complemented strain (Compl; crossed circles) were analyzed for average numbers of apoptotic cells that were positive for caspase-3 from six high power fields (400x magnification) per mouse. Uninfected controls received vehicle only (none, white diamonds). Medians (black bars), significance levels (*p*-values) determined by the Kruskal–Wallis test and Dunn’s post-correction and the numbers of analyzed mice (in parentheses) are indicated. Data were pooled from four independent experiments.

Secretion of gamma-interferon was determined in the (a) liver, (b) kidney, and (c) lung biopsies of mice on d6 following infection with *C.jejuni* 81–176 WT (black circles), Δ*pepP* (white circles), or *pepP* complemented strain (Compl; crossed circles), and of uninfected control mice (none, white diamonds). Medians (black bars), significance levels (*p*-values) determined by the Kruskal–Wallis test and Dunn’s post-correction and the numbers of analyzed mice (in parentheses) are indicated. Data were pooled from four independent experiments.

Serum levels of pro-inflammatory mediators in mice on d6 following infection either with WT (black circles), Δ*pepP* (white circles), or *pepP* complemented strain (Compl; crossed circles), and control mice (none, white diamonds) were determined for (a) IFN-γ, (b) TNF, (c) MCP-1, and (d) IL-6 concentrations. Medians (black bars), significance levels (*p*-values) determined by the Kruskal–Wallis test and Dunn’s post-correction and the numbers of analyzed mice (in parentheses) are indicated. Data were pooled from four independent experiments.

## Discussion

*Campylobacter jejuni* infections are associated with strong inflammatory responses resulting in tissue damage within the human gut, which is mainly attributed to bacteria invading epithelial cells and traversing the intestinal barrier upon infection. *C. jejuni* is able to enter the lamina propria and reach the bloodstream and may travel to other organs, such as liver, spleen, and mesenteric lymph nodes. However, the molecular mechanisms and required bacterial factors are not fully understood. Peptidases produced by *C. jejuni* are important for bacterial growth and sequestering of peptides and/or amino acids in the gastrointestinal tract of the mammalian host,^29^ and several putative peptidases and proteases have been recognized in so far published *C. jejuni* genomes.^30,31^ One *C. jejuni* protease directly participating in pathogenicity is the secreted serine protease HtrA, which enables the bacteria to transmigrate effectively across polarized intestinal epithelial cells using the paracellular route.^27,32^ Other putative proteases and peptidases have also been linked to the pathogenicity of the organism, for instance, ClpP (Cj0192 c),^33^ CJJ81176_1086 (Cj1068),^34^ Pgp1 (Cj1345 c; CJJ81176_1344),^35^ Cj1365^36^ and serine peptidase Cj0511; the latter was shown to be essential for chicken colonization.^26^ Outer membrane vesicles (OMVs) shed by *C. jejuni* can contain combinations of Cj0511, Cj1365 c, and HtrA.^37^ Interestingly, OMVs produced in the presence of the bile salt taurocholic acid exhibited increased protease activity and were shown to have a crucial role for *C. jejuni* invasion and transmigration *in vitro*.^38^ Although this underlines the putative function of proteases in *C. jejuni* pathogenicity, an *in vivo* role in virulence of these proteases (except for HtrA) has not yet been demonstrated, for instance by use of a suitable animal model. Here, we identified the peptidase PepP by casein zymography and mass spectrometry, and demonstrate that inactivation of its gene in *C. jejuni* strain 81–176 resulted in a marked decrease of virulence during infection of microbiota-depleted IL-10^−/-^ mice.

Our present study revealed that *pepP* gene deficiency did not compromise the colonization properties of *C. jejuni* toward the large intestine in microbiota-depleted IL-10^−/-^ mice, but it did decrease numbers in the ileum. We have previously reported that mutants, in which the entire *htrA* gene or its serine protease active site had been inactivated, colonize the colon as well as WT bacteria.^39,40^ Whereas mice infected with WT or *pepP* complemented bacteria were suffering from severe symptoms including wasting and bloody diarrhea within 1 week post-infection, the induced disease was much less pronounced upon *pepP* gene deletion, as indicated by lower median clinical scores. Of note, the variability of macroscopic outcome in the Δ*pepP* infected cohort was relatively high, given that some mice did not exhibit any overt symptoms, whereas others presented with severe disease. It needs to be taken into consideration that the symptoms of campylobacteriosis are the sum of distinct pathophysiological sequelae of *C. jejuni* infection. The orchestrated interplay between known and as yet unknown virulence factors with host factors defines the disease outcome. The overall better clinical outcome of infections with the *C. jejuni* 81–176 *pepP* mutant compared to the WT and *pepP* complemented strain was accompanied with immunopathological sequelae that provide insights in the disease such as (i) fewer induced apoptotic epithelial cells, (ii) more pronounced proliferative and regenerative colonic epithelial cell responses, (iii) a weaker innate immune response, combined with comparable adaptive immune cell responses in the large intestinal mucosa and lamina propria, and (iv) less pronounced secretion of pro-inflammatory mediators in the intestinal tract. The immunopathological responses following epithelial damage make up the inflammatory nature of the disease, which is triggered by factors for which an intact *pepP* gene is required. In this view, it is not surprising that the impact of PepP in murine campylobacteriosis was not restricted to the intestinal tract but could also be demonstrated in other organs and even in systemic responses. In support of these observations, we have previously reported that loss of *C. jejuni* HtrA was accompanied by less distinct pro-inflammatory sequelae in liver and kidneys of infected mice.^40^

Although we have pinpointed here an important role of PepP in campylobacteriosis in mice, its exact role in this scenario or the targeted substrates of this protease are yet unknown. The finding that apoptosis in the colon is significantly diminished in mice infected with the *pepP* mutant (as compared to mice infected with the WT or *pepP* complemented strain) provides strong evidence that the epithelial layer is the first and main target of PepP in the intestines. Interestingly, similar to other gut microbiome bacteria, amino acid- and peptide-catabolism promote the proliferation of *C. jejuni*.^41–44^ In this regard, *C. jejuni* differs substantially from various enteritis-associated pathogens of the Enterobacteriaceae in the intestine which utilize sugars as substrates for energy and carbohydrate metabolism.^45^ Instead, *C. jejuni* preferably catabolizes amino acids and oligopeptides released by proteolytic enzymes from digested proteins, which are available in the host gut. Indeed, it has been shown in a mouse model that *C. jejuni* mutated in the carboxyl-terminal protease CtpA, in the zinc metalloendopeptidase PepF, or in a member of the C26 endopeptidase family CJJ81176_1416 resulted in a strong decrease of intestinal colonization.^46^ Except for the hydrolytic activity of the gamma-glutamyltranspeptidase (GGT) in *C. jejuni* 81–176,^42^ a defined role of other proteases in the acquisition of amino acid nutrients by the bacterium has not yet been reported. Such function is less likely for the peptidase PepP (and for HtrA), since their inactivation did not impair the colonization capacity. This suggests that either their peptidase function is highly redundant, or it is not involved in nutrient sequestering. More likely, these enzymes ensure specific virulence functions. One possibility is that PepP may be secreted in the extracellular environment just like HtrA is, and their combined action more effectively targets host cell proteins, such as in the epithelial junctional complexes, but this still needs to be established.

## Material and methods

### Casein zymography and mass spectrometry

Protein extracts of *C. jejuni* strain 81–176 (lysed bacterial pellet) were prepared and subjected to casein zymography as previously described.^47^ A negatively stained protein band, indicative of protease activity, was excised from the gel and digested with the ProteoExtract All-In-One Trypsin Digestion Kit (Calbiochem, Gibbston, NJ). Resulting peptides were separated by capillary-reversed phase high-pressure liquid chromatography directly coupled to a Quadrupole-Time of Flight mass spectrometer (QTof Ultima Global, Waters, Milford, MA) and analyzed as described earlier.^48^ Obtained mass data were processed and analyzed with Protein Lynx Global Server version 2.2.5 (Waters, Milford, MA).

### Gene identification, assessment of conservation, and phylogenetic analysis

The gene encoding the peptidase was identified by searches with the obtained trypsin fragments using the TrEMBL database. After identification of the gene in the genome of *C. jejuni* strain 81–176 (NC_008787.1), its protein sequence was used as the BlastP query at NCBI. First, a BlastP search with the gene from *C. jejuni* strain 81–176 as the query was performed and when possible, protein hits found by >10 members were retrieved. For *Campylobacter* species not producing hits with >10 members, single hits were recorded that represented the highest and lowest identity to the complete query sequence. The first hit to a species not belonging to the *Campylobacter* genus was also selected. A multiple alignment was produced with Muscle and a phylogenetic ML tree was constructed with IQ-Tree (Blosum62) with ultrafast bootstrap analysis. Three PFam domains were downloaded from the PFam website which were used for comparison with the *C. jejuni* 81–176 gene sequence. Conservation based on a multiple alignments of the closest homologs in *C. jejuni, C. coli*, and *C. hepaticus* was included.

### Gene inactivation and genetic complementation

The *pepP* gene was inactivated by homologous gene recombination in *C. jejuni* 81–176. For this purpose, the 1,791 bp gene sequence was PCR amplified with the primers 5ʹ-ATGAGTATTTACAAACAAAGAGTA-3 and 5ʹ-TTAAAAGATGGCTTTTGTTCTTTTT-3ʹ and subsequently cloned into plasmid pGEM-Teasy (Promega). A 122 bp sequence from the middle of the gene was excised by restriction enzymes *Hind*III and *Swa*I, and replaced by insertion of a kanamycin gene cassette (AphA-3). This construct was electroporated into the WT strain. Genetic complementation of the ∆*pepP* mutant with the WT *pepP* gene was performed as described previously.^24^ Genetic mutants were selected on agar plates containing kanamycin and correct insertion of the cassette was confirmed by PCR and sequencing of the amplicons using standard procedures.

### Bacterial cultivation for murine infection

For *C. jejuni* infection, stock solutions of respective *C. jejuni* strains that had been stored at −80°C were thawed, aliquots streaked onto karmali agar (Oxoid, Wesel, Germany) and incubated in a microaerophilic atmosphere at 37°C for 48 hours. Immediately before peroral infection of mice, bacteria were harvested in sterile PBS or Mueller Hinton (MH) broth (both from Oxoid) to a comparable final inoculum of 10^9^ bacterial cells per *C. jejuni* strain.

### Microbiota depleted IL-10^−/-^ murine infection model

The applied animal model is described in detail elsewhere.^28^ In order to overcome natural murine colonization resistance toward *C. jejuni*, IL-10^−/-^ mice (in a C57BL/6 j background) were first subjected to broad-spectrum antibiotic treatment.^49^ Subsequent removal of the commensal gut microbiota not only eradicates potential bacterial-related colitogenic stimuli but additionally overrides physiological colonization resistance allowing colonization by *C. jejuni* following oral application.^49,50^ In brief, 3-week-old female and male littermate mice received a 10-week course of broad-spectrum antibiotic treatment of ampicillin plus sulbactam (1 g/L; Ratiopharm, Germany), vancomycin (500 mg/L; Cell Pharm, Germany), ciprofloxacin (200 mg/L; Bayer Vital, Germany), imipenem (250 mg/L; MSD, Germany) and metronidazole (1 g/L; Fresenius, Germany). Two days before infection the antibiotic treatment was terminated. The then 3-month-old, sex-matched animals were challenged perorally with 10^9^ CFU of the *C. jejuni* parental WT strain 81–176, the isogenic Δ*pepP* mutant or the *pepP* complemented strain in 0.3 mL phosphate-buffered saline (PBS, Gibco, life technologies, UK) on d 0 and 1 by gavage. A non-infected control group received sterile PBS but was otherwise treated identically. The animals were maintained in a sterile environment with *ad libitum* autoclaved food and drinking water and handled under strict aseptic conditions throughout the experiment to avoid contamination.

Feces were sampled daily and quantitatively analyzed for the presence of *C. jejuni* by serial dilutions and agar plate enumerations as described previously.^50^

### Clinical conditions of mice

The clinical conditions of mice were assessed immediately before and after *C. jejuni* infection on a daily basis until termination of the experiment by a standardized cumulative clinical score (maximum 12 points) addressing the clinical aspect (0: normal; 2: ruffled fur, less locomotion; 4: isolation, severely compromised locomotion, pre-final aspect), the stool consistency (0: formed feces; 2: pasty feces; 4: liquid feces), and the abundance of blood in fecal samples (0: no blood; 2: microscopic detection of blood by the Guajac method using Haemoccult, Beckman Coulter/PCD, Germany; 4: macroscopic blood visible) as described earlier.^40^

### Sampling procedures and immunohistochemistry

On d6 p.i., the mice were sacrificed by isoflurane inhalation (Abbott, Germany) and blood generated by cardiac puncture for serum measurements. *Ex vivo* biopsies from colon, ileum, MLN, liver, kidneys, and lungs and were obtained under sterile conditions. Parallel samples of the intestinal and extraintestinal tract were taken from each mouse for microbiological, immunohistopathological, and immunological analyses. To assess gastrointestinal pathogen loads luminal contents from the stomach, duodenum, ileum, and colon were squeezed into a tube containing sterile PBS and plated in serial dilutions onto solid media as described previously.^50^ The weight of respective luminal sample was determined by the difference of weights of each tube after and before adding the luminal content (in g). The organ biopsies were directly fixed within 5% formalin, embedded in paraffin, and analyzed immunohistochemically as described earlier.^51,52^ For the detection and quantitation of apoptotic epithelial cells, we used primary antibodies directed against cleaved caspase 3 (Asp175, Cell Signaling, Beverly, MA, USA, 1:200); proliferating and regenerating epithelial cells were identified using Ki67-specific antibodies (TEC3, Dako, Denmark, 1:100). Macrophages and monocytes were visualized with antibodies directed against F4/80 (# 14–4801, clone BM8, eBioscience, San Diego, CA, USA, 1:50), while T lymphocytes, regulatory T cells, and B lymphocyte were detected with primary CD3 (#N1580, Dako, 1:10), FOXP3 (FJK-16 s, eBioscience, 1:100), and B220 (eBioscience, SanDiego, CA, USA, 1:200) antibodies, respectively. Positively stained cells were counted using blinded samples within at least six high power fields (0.287 mm^2^, 400 x magnification) by light microscopy.

### Pro-inflammatory mediator detection

Colonic and ileal tissue samples were cut longitudinally and washed in PBS after which strips of 1 cm^2^ were incubated for 18 h at 37°C in 24-flat-bottom well plates (Nunc, Germany) containing 500 μL serum-free RPMI 1640 medium (Gibco, life technologies, UK) with 100 U/mL penicillin and 100 µg/mL streptomycin (PAA Laboratories, Germany). Biopsies of MLN (3 single lymph nodes), liver (1 cm^3^), one-half kidney (cut longitudinally), and one lung were treated likewise. After incubation, culture supernatants were harvested and analyzed for the presence of TNF, IFN-γ, MCP-1, and IL-6 by the Mouse Inflammation Cytometric Bead Array (BD Biosciences, Germany) on a BD FACSCanto II flow cytometer (BD Biosciences). Systemic pro-inflammatory cytokines were measured in serum samples. Nitric oxide (NO) concentrations were determined as described earlier.^49^

### Western blotting

Pelleted bacterial cells were mixed with an equal amount of Laemmli buffer and boiled for 5 minutes.^24^ The proteins were separated by SDS-PAGE on 10% polyacrylamide gels followed by blotting on PVDF membrane (Immobilon-P, Merck Millipore, Darmstadt/Germany). The membranes were blocked in TBS-T (140 mM NaCl, 0.1% Tween-20, 25 mM Tris-HCl pH 7.4) with 3% BSA for 1 h at room temperature before the addition of the antibodies. Rabbit polyclonal α-HtrA antibodies were described previously.^25^ Rabbit polyclonal α-M24 (PepP) antibodies were generated by immunizing rabbits using the conserved M24-derived peptide C-LIDSGGQYKNGTTDI. The specificity of the latter antibody was approved by Dotblots using peptide LIDSGGQYKNGTTDI and various non-M24 control peptides (data not shown). Immunization was performed according to the German Tierschutzgesetz and Tierschutz-Versuchsverordnung as implementation of the EU directive 2010/63/EU. The corresponding protocol has been approved by Landesamt für Landwirtschaft, Lebensmittelsicherheit und Fischerei Mecklenburg-Vorpommern (LALLF M-V, Rostock/Germany). The antibodies were affinity-purified using a standardized protocol of the company (Biogenes GmbH, Berlin/Germany). As secondary antibodies, we used horseradish peroxidase-conjugated α-rabbit polyvalent goat immunoglobulins (Thermo Fisher Scientific, Massachusetts, USA). The ECL Plus chemiluminescence Western Blot system was applied for immunostaining (GE Healthcare).

## Statistical analysis

Medians and levels of significance were determined using Mann–Whitney test (GraphPad Prism v7, USA) for pairwise comparisons of not normally distributed data, and using the one-sided ANOVA test with Tukey post-correction or the Kruskal–Wallis test with Dunn’s correction for multiple comparisons as indicated. Two-sided probability (p) values ≤0.05 were considered significant. All experiments were performed in four replicates.

## Supplementary Material

Supplemental Material

## Data Availability

The datasets supporting the conclusions of this article are included within the article and its additional files. Materials are available upon request.
