# Supplementary material for: Peptidase PepP is a novel virulence factor of Campylobacter jejuni contributing to murine campylobacteriosis
Source: Gut Microbes. 2020 Jun 25;12(1):1770017. doi: 10.1080/19490976.2020.1770017 (PMC7524167; doi:10.1080/19490976.2020.1770017)

A

Apoptotic Cells (Casp3+) - COLON

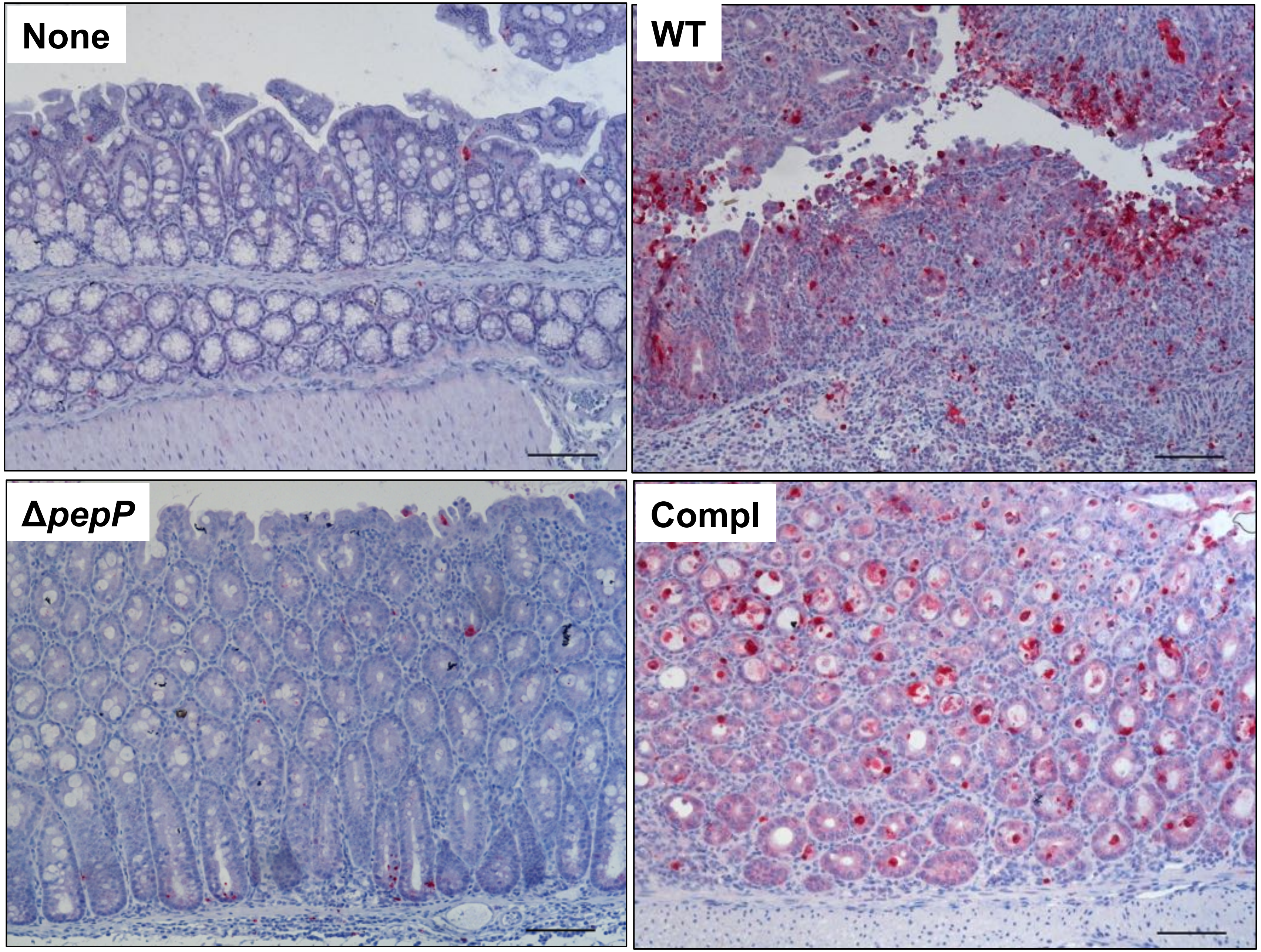

B

Proliferating Cells (Ki67+) - COLON

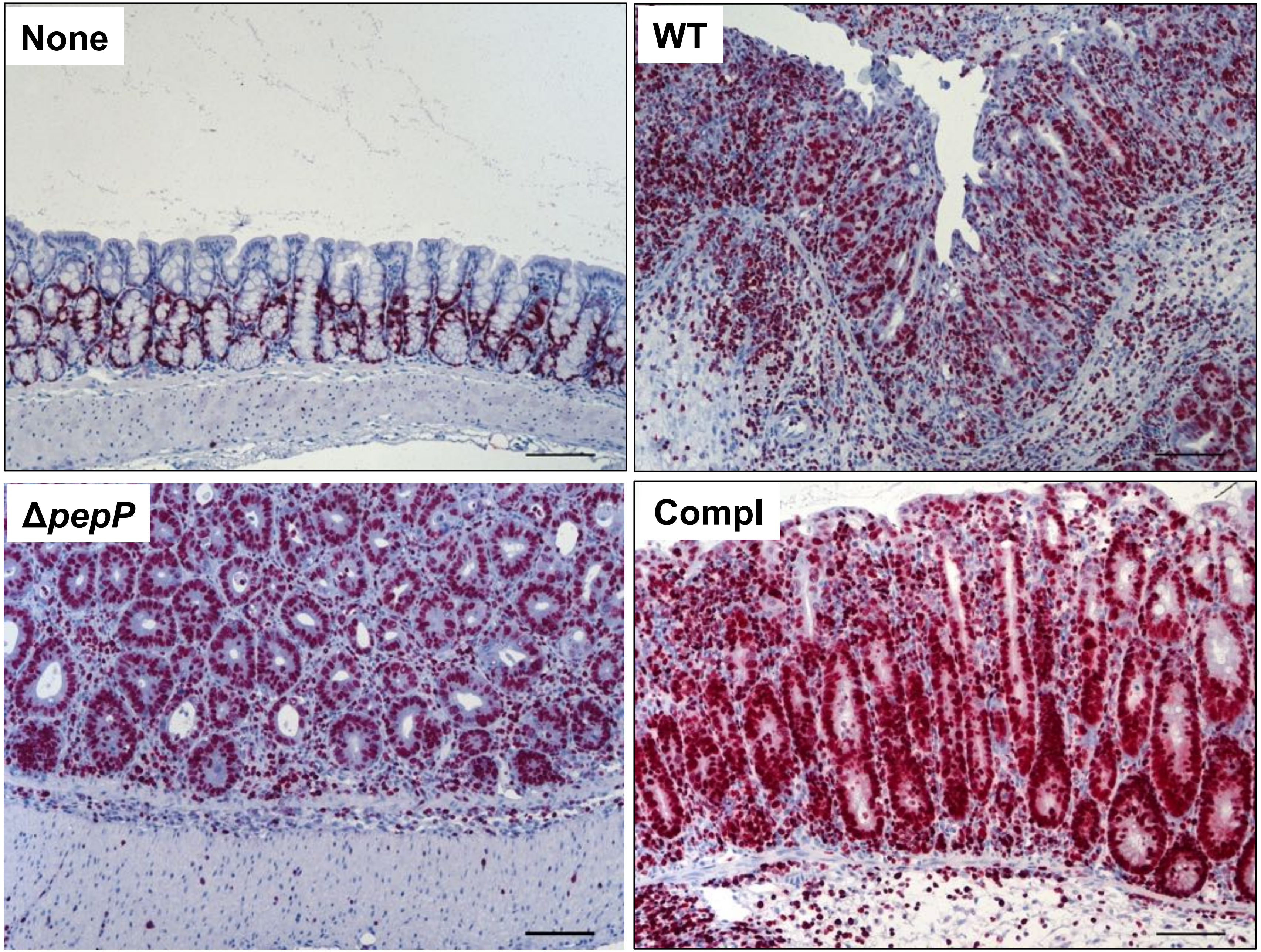

C

Macrophages / Monocytes (F4/80+) - COLON

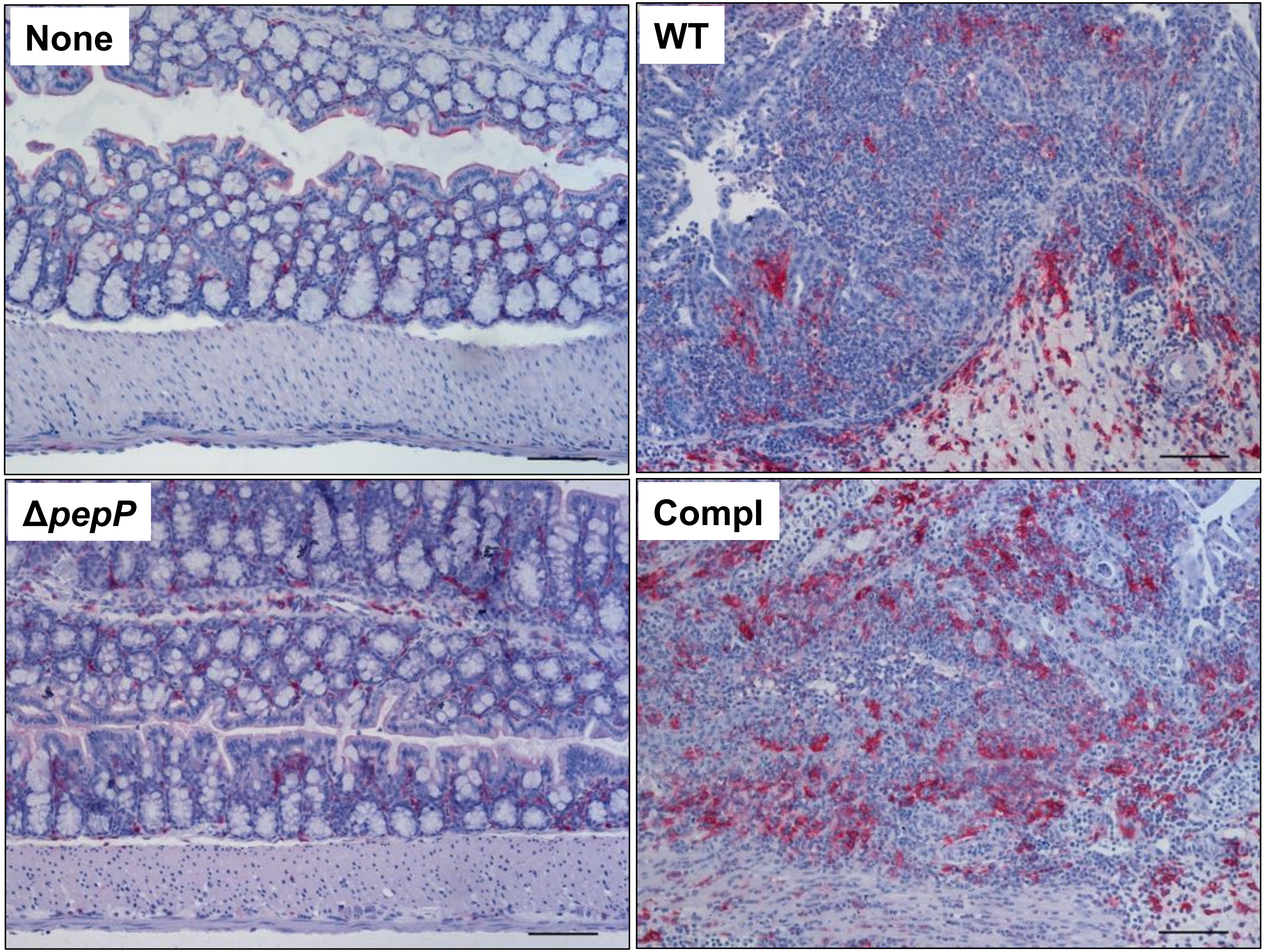

D

T Lymphocytes (CD3+) - COLON

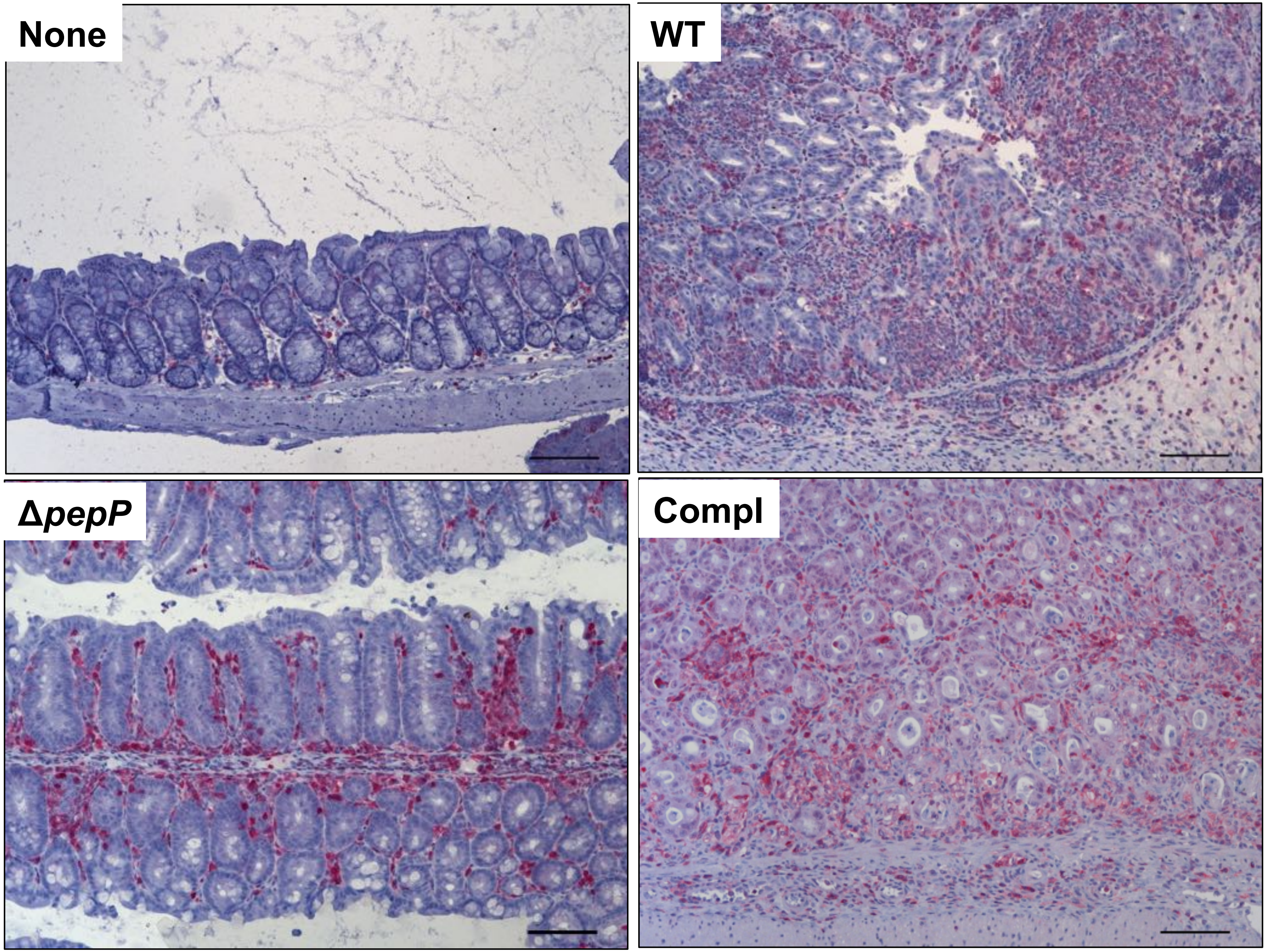

E

Regulatory T Cells (FOXP3+) - COLON

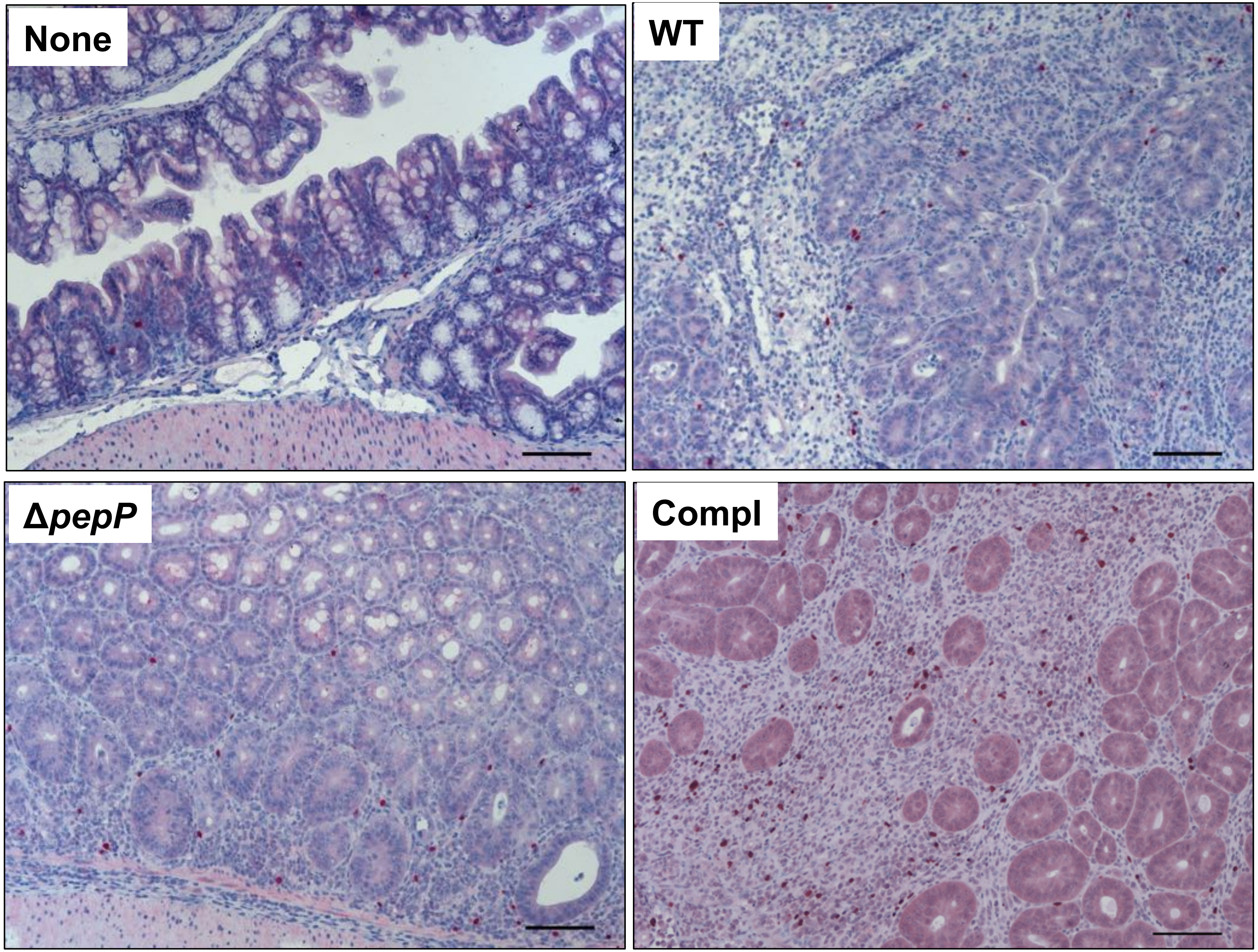

F

B Lymphocytes (B220+) - COLON

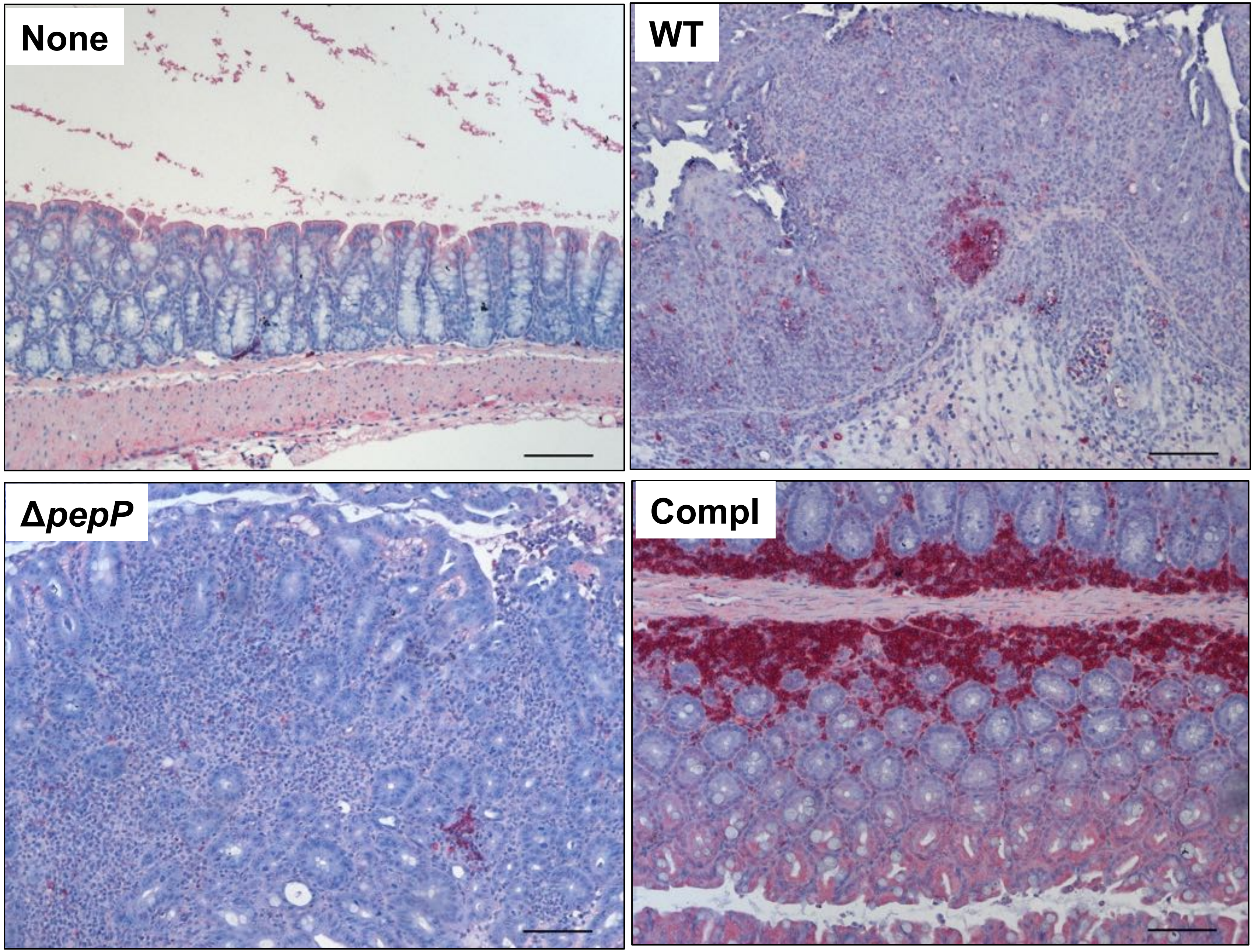

Supplement: Supplemental Material [file KGMI_A_1770017_SM3114.zip › FigS5_REVISED_COLON_PICS_020420.pdf]
