# Supplementary material for: Peptidase PepP is a novel virulence factor of Campylobacter jejuni contributing to murine campylobacteriosis
Source: Gut Microbes. 2020 Jun 25;12(1):1770017. doi: 10.1080/19490976.2020.1770017 (PMC7524167; doi:10.1080/19490976.2020.1770017)
Supplement: Supplemental Material [file KGMI_A_1770017_SM3114.zip › Supplementary material.docx]

**Supplemental Material:**

**Figure S1: Logoplots of the three PFam domains present in PepP.** The PFam domains were downloaded from the PFam website. The plots were aligned with the PepP protein sequence of *C. jejuni* strain 81-176 and the conservation is shown within all *C. jejuni*, *C. coli* and *C. hepaticus* genes for (**A**) PF01321, Creatinase N1, representing amino acid 7 to 145 of PepP; (**B**) PF16189, Creatinase N2, representing amino acid 146 to 301; and (**C**) PF16188, Peptidase M24-C, spanning amino acid 533 to 595. The size of the amino acids shown in the plots is proportional to their conservation amongst a large number of protein entries.  In the aligned sequence, strongly conserved amino acids in the logoplot are similarly colored and connected with a line.

**Figure S2: Phylogenetic tree of PepP in *Campylobacter* species.** The protein found in *C. jejuni* strain 81-176 is identical to that of 16 other *C. jejuni* entries currently present in GenBank. This sequence was used as the query to find homologs by BlastP. Homologs covering >80% of the query sequence with >50% identity were retrieved. An alignment was created with Muscle and genetic tree (Maximum Likelihood) was constructed with IQ-Tree (Blosum62) with ultrafast bootstrap analysis. The closest homologs were identified in *C. jejuni*, *C. coli* and *C. hepaticus*, with more distant homologs present in four other *Campylobacter* species. The tree was rooted with the most similar outlier of PepP that was identified, from *Helicobacter mesocricetorum*.

**Figure S3: Growth curves of *C. jejuni* strains used in this study.** The wild-type strain 81-176 (WT; black circles), the isogenic Δ*pepP* gene deletion mutant (red circles) and the complemented *pepP* strain (blue circles) were adjusted in liquid MH medium to an optical density (OD) of 0.1 as measured at 600 nm using an Eppendorf spectrophotometer. Growth of the bacteria was then followed in a time course for 16 hours, 24 hours, 36 hours and 48 hours. All experiments were performed in triplicates.

**Figures S4: Intestinal pathogenic colonization in *C. jejuni* Δ*pepP* infected mice over time.** Microbiota depleted IL-10^-/-^ mice were perorally infected either with **(A)** the *C. jejuni* 81-176 wild-type strain (black circles) or **(B)** the isogenic *pepP* gene deletion mutant strain (Δ*pepP*; white circles) on day (d) 0 and d1. Fecal *C. jejuni* loads were quantitatively assessed over time post-infection by culture and expressed as colony forming units per g (CFU/g). Numbers of analyzed animals are given in parentheses. Data were pooled from four independent experiments.

**Figure S5: Representative photomicrographs illustrating apoptotic, proliferative/regenerative and immune cell responses in the colon.** Paraffin sections of colon biopsies were obtained six days following infection with *C. jejuni* 81-176 wild-type (WT), Δ*pepP* or *pepP* complemented (Compl) strains and analyzed for **(A)** apoptotic epithelial cells that are positive for caspase-3, **(B)** proliferating/regenerating epithelial cells positive for Ki67, **(C)** macrophages and monocytes determined by F4/80, **(D)** CD3^+^ T lymphocytes, **(E)** FOXP3^+^ regulatory T cells, and **(F)** B220^+^ B lymphocytes in the mucosa and lamina propria. Uninfected controls received vehicle only (none). Photomicrographs (100 x magnification, scale bar 100 μm) shown are representative for four independent experiments.

**Figure S6:** **Ileal pro-inflammatory mediator secretion in *C. jejuni* Δ*pepP* infected mice.** Microbiota depleted IL-10^-/-^ mice were perorally infected either with the *C. jejuni* 81-176 WT (black circles), Δ*pepP* (white circles) or *pepP* complemented strain (Compl; crossed circles) on days 0 and 1. **(A)** IFN-γ and **(B)** TNF concentrations were measured in supernatants of ileal *ex vivo* biopsies taken on day 6 post-infection. Uninfected controls received vehicle only (none, white diamonds). Medians (black bars), significance levels (p-values) determined by the Kruskal-Wallis test and Dunn’s post-correction and the numbers of analyzed mice (in parentheses) are indicated. Data were pooled from four independent experiments.

**Figure S7: Representative photomicrographs illustrating apoptotic and proliferative/regenerative cells in extra-intestinal organs.** Paraffin sections of (**A**) liver, (**B**) kidney and (**C**) lung biopsies were obtained six days following infection with *C. jejuni* 81-176 wildtype (WT), Δ*pepP* or *pepP* complemented (Compl) strains analyzed for apoptotic cells that were positive for caspase-3. Uninfected controls received vehicle only (none). Photomicrographs (100 x magnification, scale bar 100 μm) shown are representative for four independent experiments.
