# Supplementary figures and images for: Peptidase PepP is a novel virulence factor of Campylobacter jejuni contributing to murine campylobacteriosis
Source: Gut Microbes. 2020 Jun 25;12(1):1770017. doi: 10.1080/19490976.2020.1770017 (PMC7524167; doi:10.1080/19490976.2020.1770017)

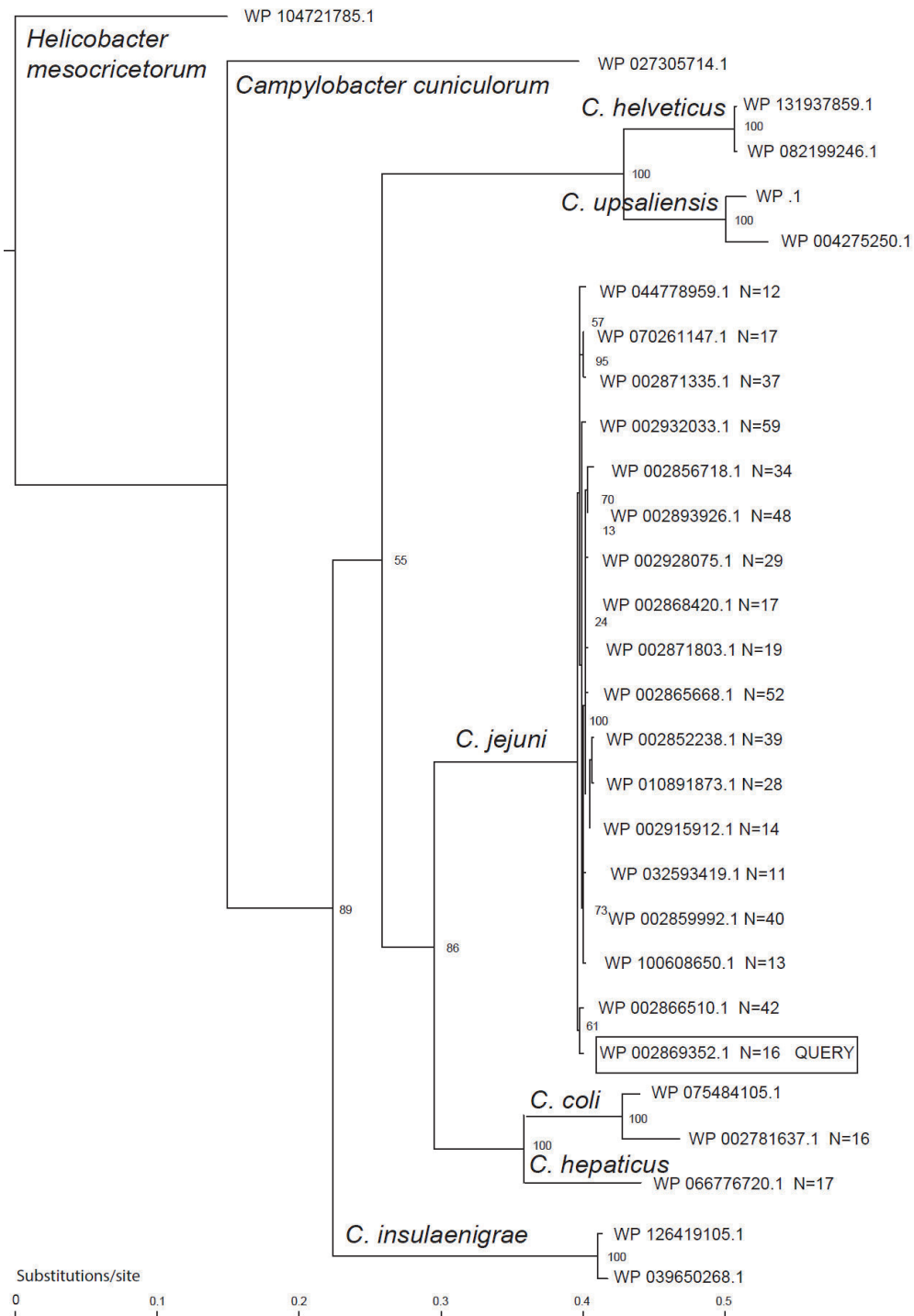

Supplement: Supplemental Material [file KGMI_A_1770017_SM3114.zip › FigS2_PepP_Campy_Tree.pdf]

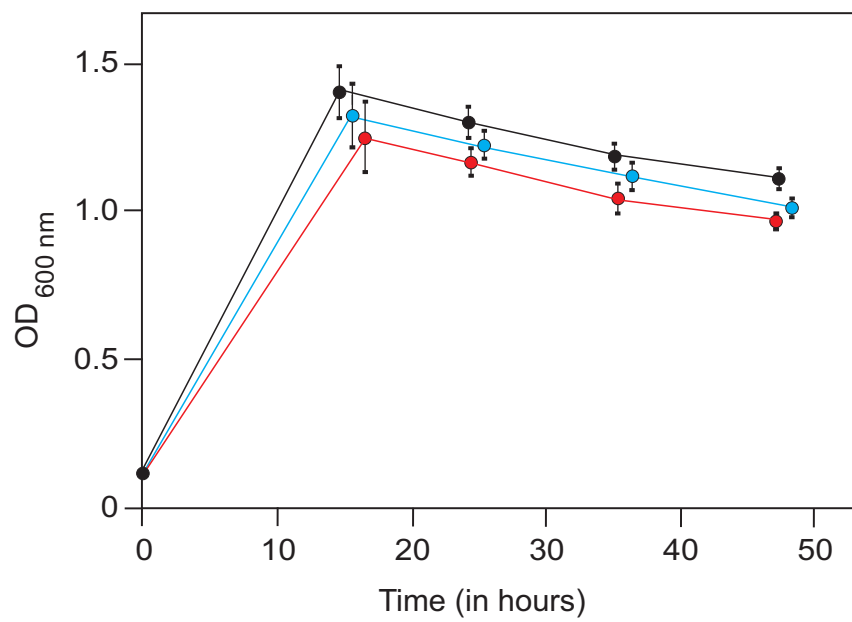

Supplement: Supplemental Material [file KGMI_A_1770017_SM3114.zip › FigS3_REVISED_Campy_Growth_310320.pdf]

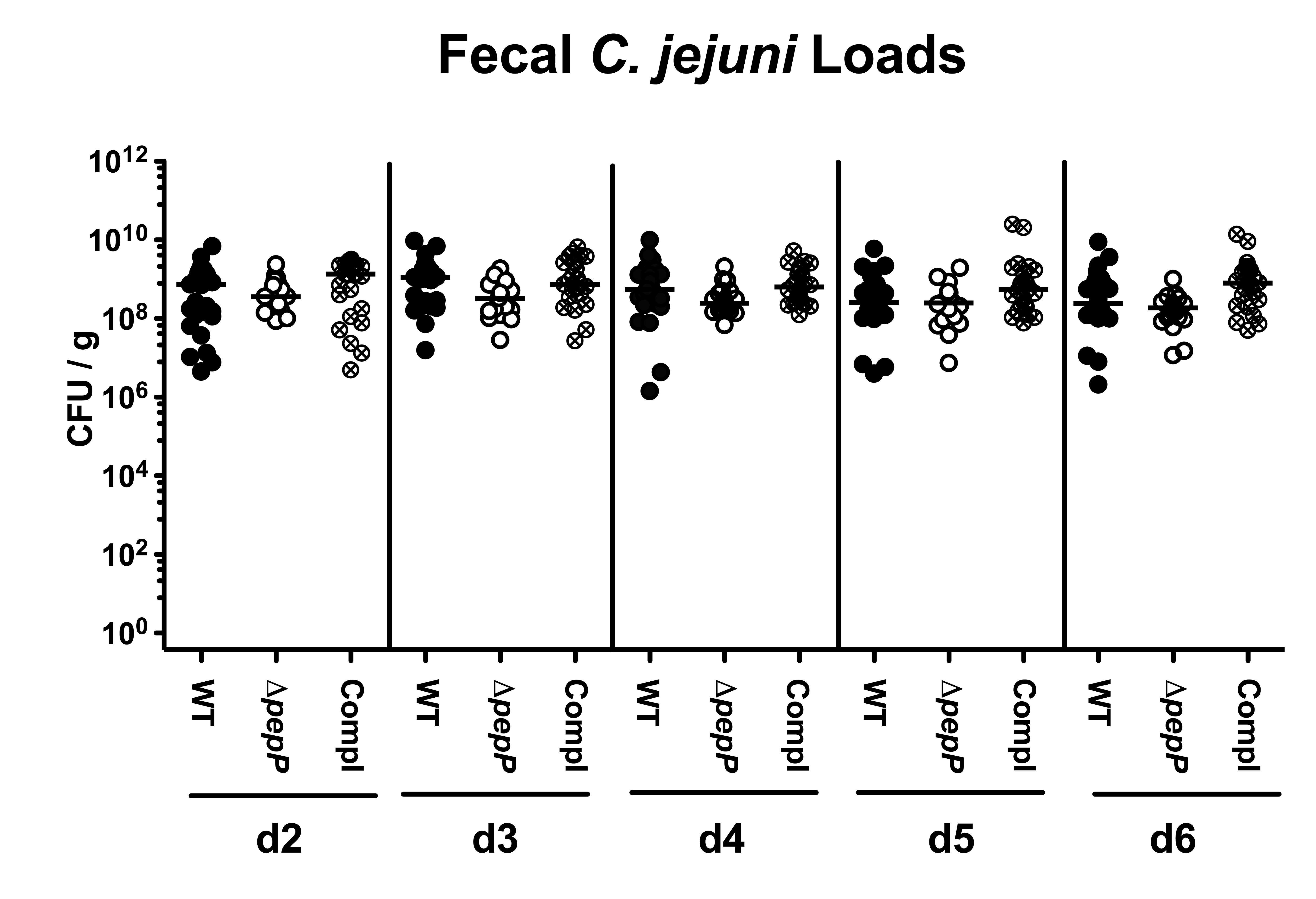

Supplement: Supplemental Material [file KGMI_A_1770017_SM3114.zip › FigS4_REVISED_Campy_Kinetik_010420.tiff]

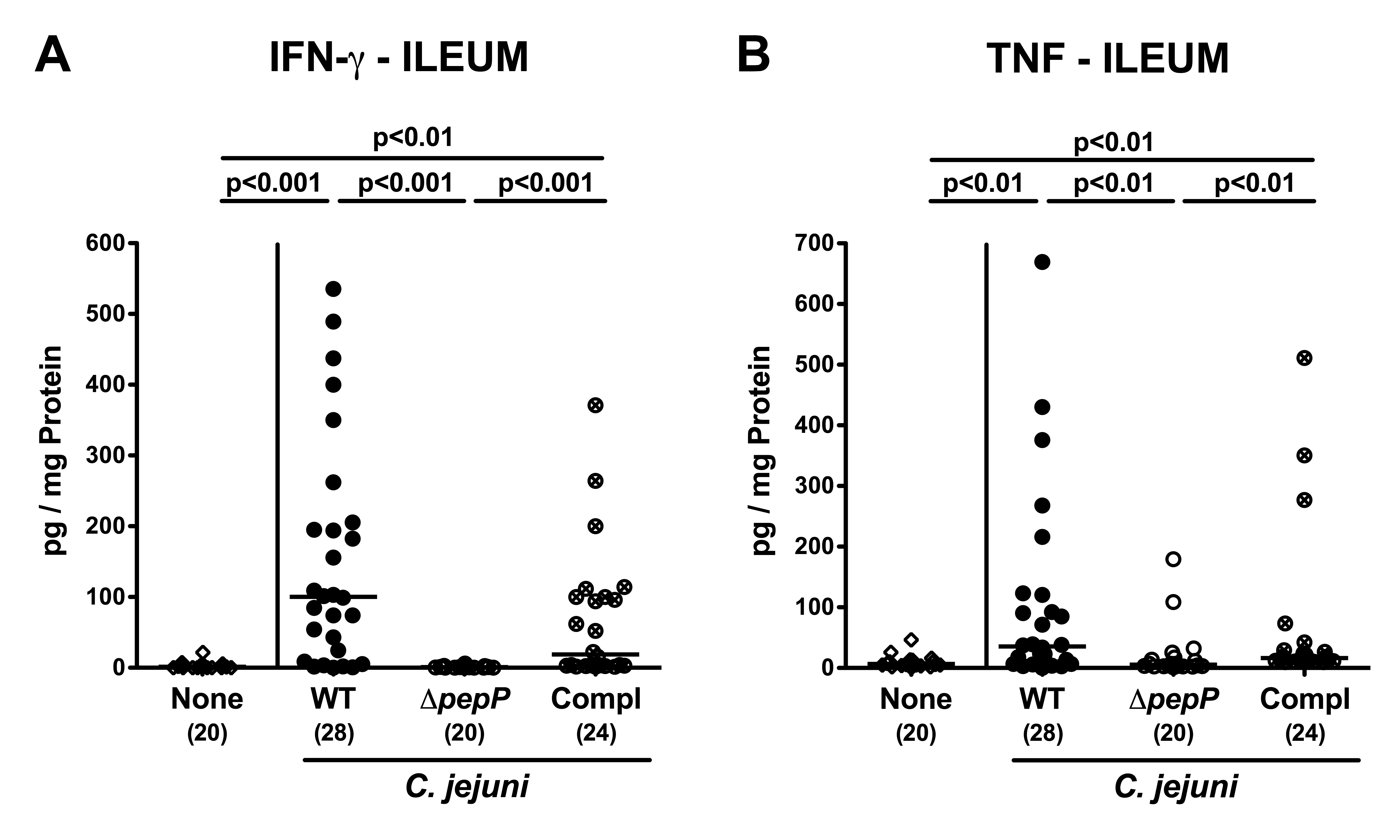

Supplement: Supplemental Material [file KGMI_A_1770017_SM3114.zip › FigS6_REVISED_Compl2_CBA_ILEUM_010420.tiff]

**A**

**Apoptotic Cells (Casp3+) - LIVER**

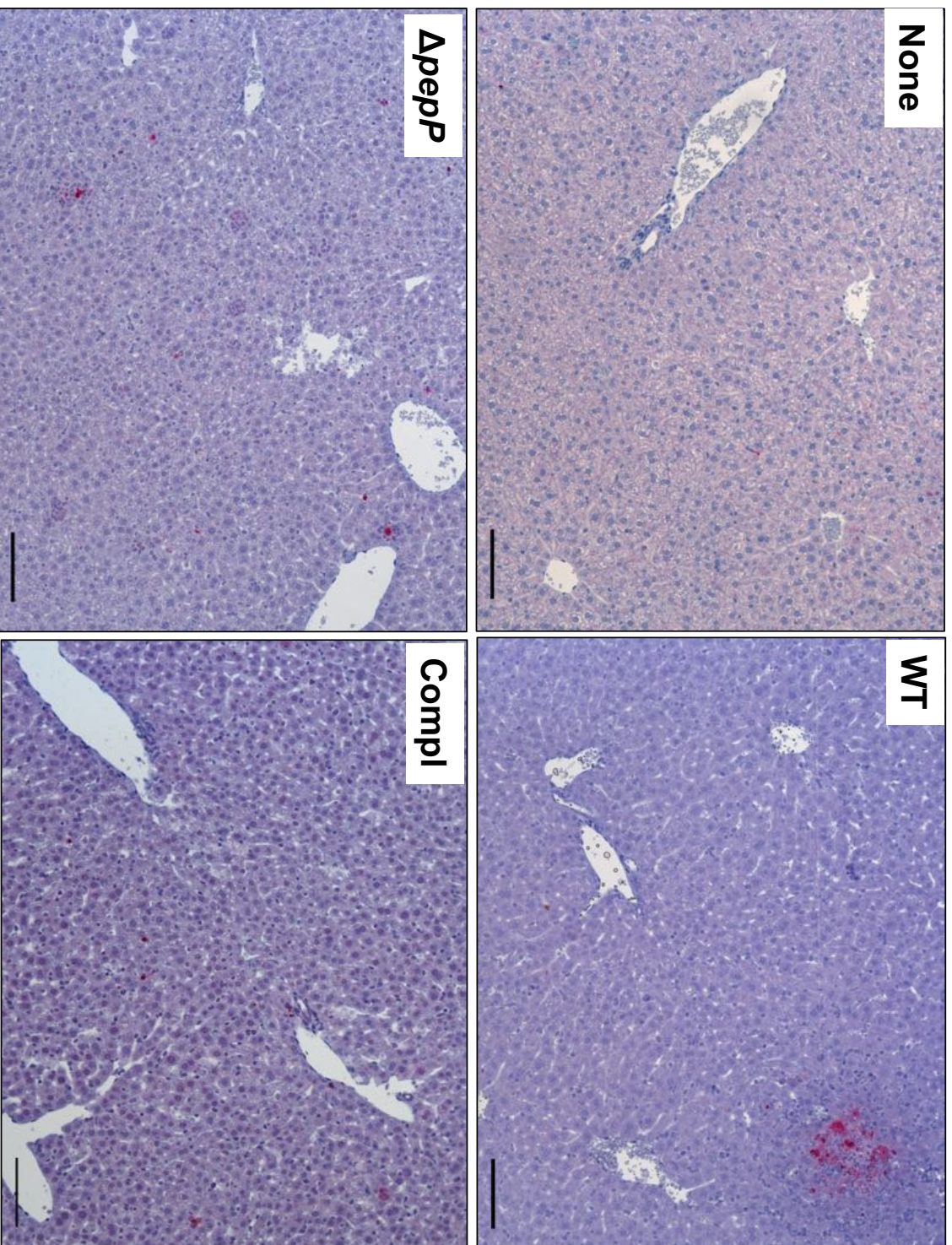

**B**

**Apoptotic Cells (Casp3+) - KIDNEY**

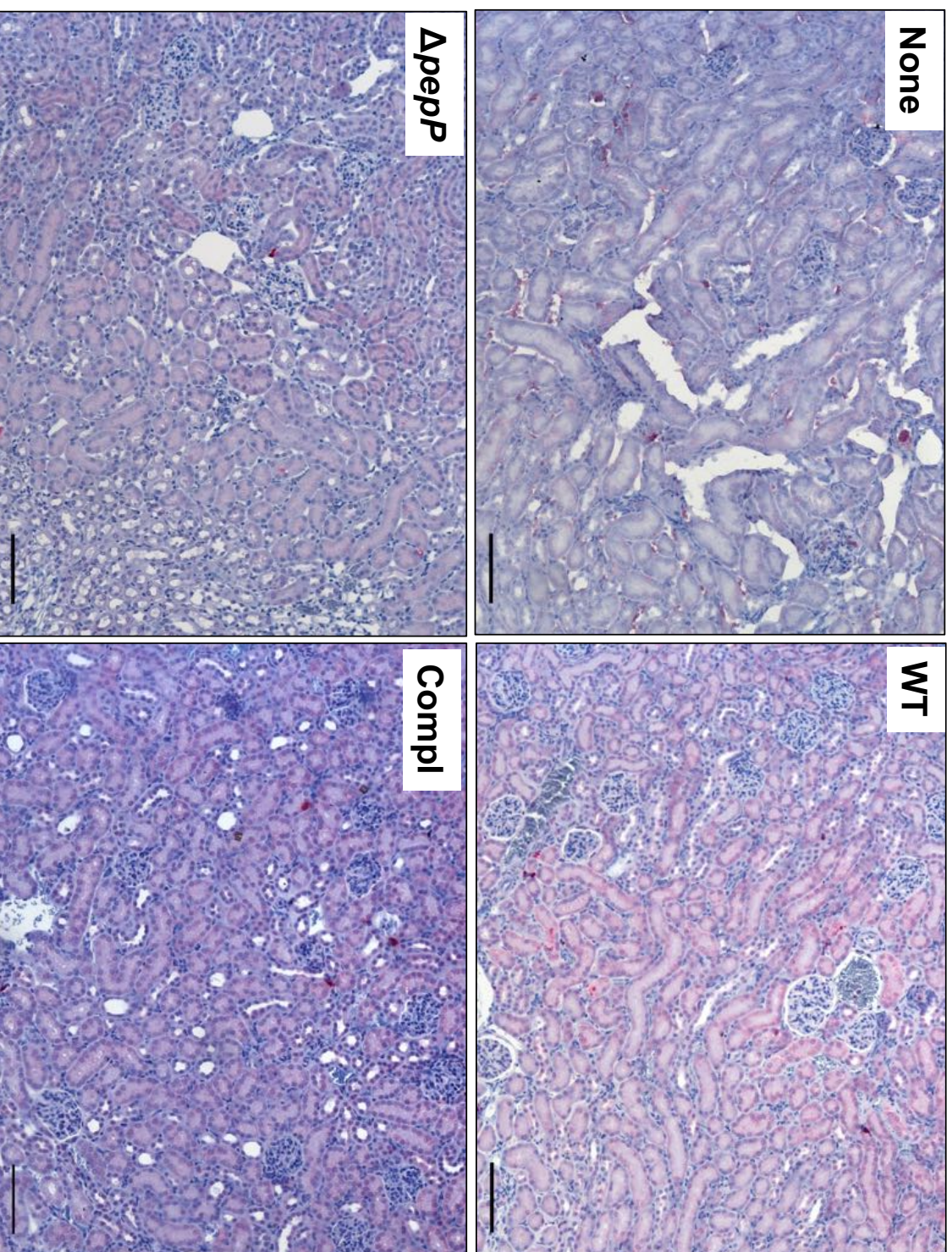

# C Apoptotic Cells (Casp3+) - LUNG

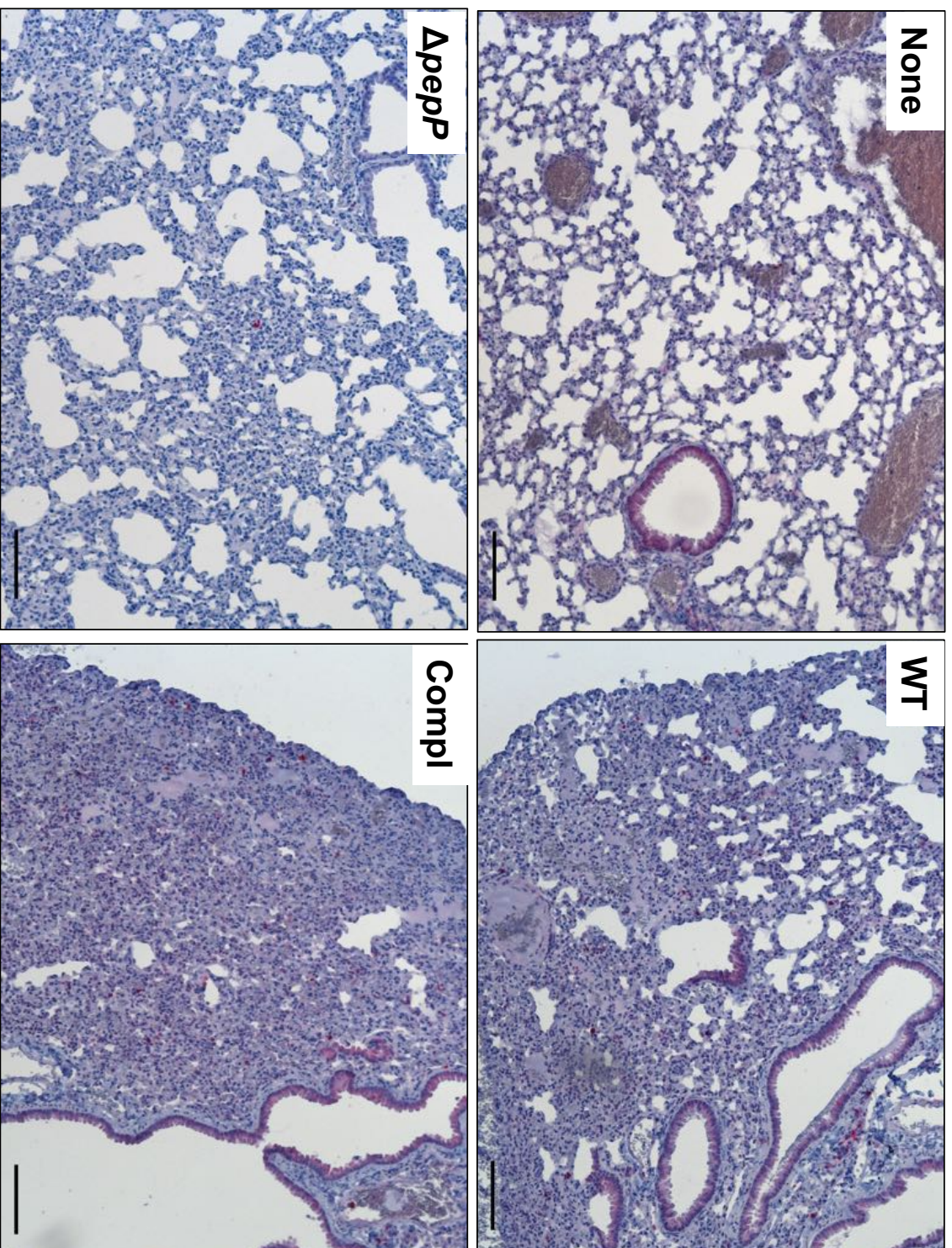

Supplement: Supplemental Material [file KGMI_A_1770017_SM3114.zip › FigS7_REVISED_Extraint_PICS_020420.pdf]
