# Supplementary material for: Peptidase PepP is a novel virulence factor of Campylobacter jejuni contributing to murine campylobacteriosis
Source: Gut Microbes. 2020 Jun 25;12(1):1770017. doi: 10.1080/19490976.2020.1770017 (PMC7524167; doi:10.1080/19490976.2020.1770017)
Supplement: Supplemental Material [file FigS1_PepP_PFam domains.pdf › FigS1_PepP_PFam domains.pdf]

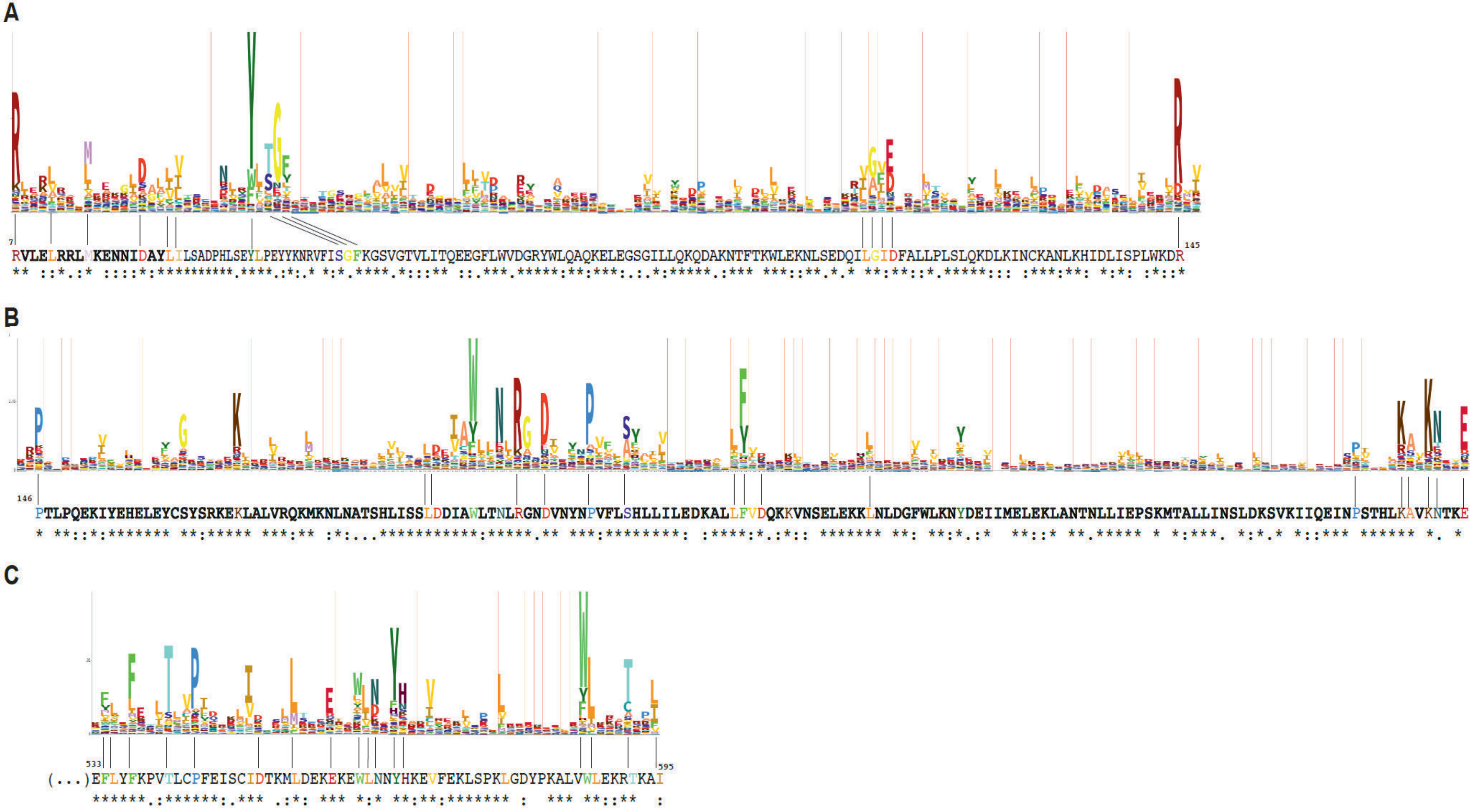
A
7
RVLELRRLMKENNIDAYLILSADPHLSEYLPEYYKNRVFISGFKGSVGTVLITQEEGFLWVDGRYWLQAQKELEGSGILLQKQDAKNTFTKWLEKNLSEDQILGIDFALLPLSLQKDLKINCKANLKHIDLISPLWKDR
145
** ::*.:* ::::*:**********.****.:*:.* *:*.****.*:::** ::.***.*****:**:***:.:.*:*****.:*** ***::**.*.* **:**::*.*****::: :***:**: *:*: :*::*
B
146
PTLPQEKIYEHELEYCSYSRKEKLALVRQKMKNLNATSHLISSLDDIAWLTNLRGNDVNYNPVFLSHLLILEDKALLFVDQKKVNSELEKKLNLDGFWLKNYDEIIMELEKLANTNLLIEPSKMTALLINSLDKSVKIIQEINPSTHLKAVKNTKE
* **::*:******* **:***** ***:** :*:...***********:*****.** ***:********: :****:*.:*:: ******* **: **:*.:* **::* **.***** * *:***. *:*.* *::*** ****** *. *
C
533
595
(...)EFLYFKPVTLCPFEISCIDTKMLDEKEKEWLNNYHKEVFEKLSPKLGDYPKALVWLEKRTKAI
******.:******:.*** .:*: *** *:* **:**:******* : *** **::** :
